# Small RNA sX13: A Multifaceted Regulator of Virulence in the Plant Pathogen *Xanthomonas*

**DOI:** 10.1371/journal.ppat.1003626

**Published:** 2013-09-12

**Authors:** Cornelius Schmidtke, Ulrike Abendroth, Juliane Brock, Javier Serrania, Anke Becker, Ulla Bonas

**Affiliations:** 1 Institute for Biology, Department of Genetics, Martin-Luther-Universität Halle-Wittenberg, Halle, Germany; 2 Loewe Center for Synthetic Microbiology and Department of Biology, Philipps-Universität Marburg, Marburg, Germany; Harvard University, United States of America

## Abstract

Small noncoding RNAs (sRNAs) are ubiquitous posttranscriptional regulators of gene expression. Using the model plant-pathogenic bacterium *Xanthomonas campestris* pv. *vesicatoria* (*Xcv*), we investigated the highly expressed and conserved sRNA sX13 in detail. Deletion of *sX13* impinged on *Xcv* virulence and the expression of genes encoding components and substrates of the Hrp type III secretion (T3S) system. qRT-PCR analyses revealed that sX13 promotes mRNA accumulation of HrpX, a key regulator of the T3S system, whereas the mRNA level of the master regulator HrpG was unaffected. Complementation studies suggest that sX13 acts upstream of HrpG. Microarray analyses identified 63 sX13-regulated genes, which are involved in signal transduction, motility, transcriptional and posttranscriptional regulation and virulence. Structure analyses of *in vitro* transcribed sX13 revealed a structure with three stable stems and three apical C-rich loops. A computational search for putative regulatory motifs revealed that sX13-repressed mRNAs predominantly harbor G-rich motifs in proximity of translation start sites. Mutation of sX13 loops differentially affected *Xcv* virulence and the mRNA abundance of putative targets. Using a GFP-based reporter system, we demonstrated that sX13-mediated repression of protein synthesis requires both the C-rich motifs in sX13 and G-rich motifs in potential target mRNAs. Although the RNA-binding protein Hfq was dispensable for sX13 activity, the *hfq* mRNA and Hfq::GFP abundance were negatively regulated by sX13. In addition, we found that G-rich motifs in sX13-repressed mRNAs can serve as translational enhancers and are located at the ribosome-binding site in 5% of all protein-coding *Xcv* genes. Our study revealed that sX13 represents a novel class of virulence regulators and provides insights into sRNA-mediated modulation of adaptive processes in the plant pathogen *Xanthomonas*.

## Author Summary

Since the discovery of the first regulatory RNA in 1981, hundreds of small RNAs (sRNAs) have been identified in bacteria. Although sRNA-mediated control of virulence was demonstrated for numerous animal- and human-pathogenic bacteria, sRNAs and their functions in plant-pathogenic bacteria have been enigmatic. We discovered that the sRNA sX13 is a novel virulence regulator of *Xanthomonas campestris* pv. *vesicatoria* (*Xcv*), which causes bacterial spot disease on pepper and tomato. sX13 contributes to the *Xcv*-plant interaction by promoting the synthesis of an essential pathogenicity factor of *Xcv*, i. e., the type III secretion system. Thus, in addition to transcriptional regulation, sRNA-mediated posttranscriptional regulation contributes to virulence of plant-pathogenic xanthomonads. To repress target mRNAs carrying G-rich motifs, sX13 employs C-rich loops. Hence, sX13 exhibits striking structural similarity to sRNAs in distantly related human pathogens, e. g., *Staphylococcus aureus* and *Helicobacter pylori*, suggesting that structure-driven target regulation via C-rich motifs represents a conserved feature of sRNA-mediated posttranscriptional regulation. Furthermore, sX13 is the first sRNA shown to control the mRNA level of *hfq*, which encodes a conserved RNA-binding protein required for sRNA activity and virulence in many enteric bacteria.

## Introduction

The survival and prosperity of bacteria depends on their ability to adapt to a variety of environmental cues such as nutrient availability, osmolarity and temperature. Besides the adaptation to the environment by transcriptional regulation of gene expression bacteria express regulatory RNAs that modulate expression on the posttranscriptional level [1], [2]. Small regulatory RNAs (sRNAs; ∼50–300 nt) have been intensively studied in the enterobacteria *Escherichia coli* and *Salmonella* spp. and, in most cases, regulate translation and/or stability of target mRNAs through short and imperfect base-pairing (10 to 25 nucleotides) [1], [3], [4], [5]. The majority of characterized sRNAs inhibits translation of target mRNAs by pairing near or at the ribosome-binding site (RBS) [1], [6]. In addition, sRNAs can promote target mRNA translation, e. g., the sRNAs ArcZ, DsrA and RprA activate translation of sigma factor RpoS [7], [8], [9]. Regulation of multiple rather than single genes has emerged as a major feature of sRNAs affecting processes like iron homeostasis, carbon metabolism, stress responses and quorum sensing (QS) [1], [2], [6]. In numerous cases, sRNAs are under transcriptional control of two-component systems (TCS), which themselves are often controlled by sRNAs [10]. The activity and stability of most enterobacterial sRNAs requires the hexameric RNA-binding protein Hfq, which facilitates the formation of sRNA-mRNA duplexes and their subsequent degradation by the RNA degradosome [1], [11]. Hfq is present in approximately 50% of all bacterial species and acts in concert with sRNAs to regulate stress responses and virulence of a number of animal- and human-pathogenic bacteria [5], [12].

To date, little is known about sRNAs in plant-pathogenic bacteria. Only recently, high throughput RNA-sequencing approaches uncovered potential sRNAs in the plant-pathogenic α-proteobacterium *Agrobacterium tumefaciens* [13], the γ-proteobacteria *Pseudomonas syringae* pv. *tomato* [14] and *Xanthomonas campestris* pv. *vesicatoria* (*Xcv*) [15], [16]. Additional studies identified four and eight sRNAs in *X. campestris* pv. *campestris* (*Xcc*) and *X. oryzae* pv. *oryzae* (*Xoo*), respectively [17], [18]. So far, only few sRNAs of plant-pathogenic bacteria were characterized with respect to potential targets. Examples include the *A. tumefaciens* antisense RNA RepE and the sRNA AbcR1, which regulate Ti-plasmid replication and the expression of ABC transporters, respectively [19], [20]. RNAs involved in virulence of plant-pathogenic bacteria were so far only reported for *Erwinia* spp. and *Xcv*. In *Erwinia*, the protein-binding RNA RsmB modulates the activity of the translational repressor protein RsmA, which impacts on QS, the production of extracellular enzymes and virulence [21], [22], [23]. In *Xcv*, sX12 was reported to be required for full virulence [16].

Xanthomonads are ubiquitous plant-pathogenic bacteria that infect approximately 120 monocotyledonous and 270 dicotyledonous plant species, many of which are economically important crops [24], [25]. These pathogens, usually only found in association with plants and plant parts, differ from most other Gram-negative bacteria in their high G+C content (∼65%), and high numbers of TonB-dependent transporters and signaling proteins [26]. Pathogenicity of most *Xanthomonas* spp. and other Gram-negative plant- and animal-pathogenic bacteria relies on a type III secretion (T3S) system which translocates bacterial effector proteins into the eukaryotic host cell [27], [28]. In addition, other protein secretion systems, degradative enzymes and QS regulation contribute to virulence of *Xanthomonas* spp. [29], [30].

One of the models to study plant-pathogen interactions is *Xcv*, the causal agent of bacterial spot disease on pepper and tomato [31], [32]. The T3S system of *Xcv* is encoded by the *hrp*− [hypersensitive response (HR) and pathogenicity] gene cluster and translocates effector proteins into the plant cell where they interfere with host cellular processes to the benefit of the pathogen [29], [33], [34]. The mutation of *hrp*-genes abolishes bacterial growth in the plant tissue and the induction of the HR in resistant plants. The HR is a local and rapid programmed plant cell death at the infection site and coincides with the arrest of bacterial multiplication [33], [35], [36]. The expression of the T3S system is transcriptionally induced in the plant and in the synthetic medium XVM2, and is controlled by the key regulators HrpG and HrpX [37], [38], [39], [40]. The OmpR-type regulator HrpG induces transcription of *hrpX* which encodes an AraC-type activator [39], [41]. HrpG and HrpX control the expression of *hrp*, type III effector and other virulence genes [16], [29], [40], [42], [43]. Recently, dRNA-seq identified 24 sRNAs in *Xcv* strain 85-10, expression of eight of which is controlled by HrpG and HrpX, including the aforementioned sX12 sRNA [15], [16].

In this study, we aimed at a detailed characterization of sX13 from *Xcv* strain 85-10, which is an abundant and HrpG-/HrpX-independently expressed sRNA [16]. Using mutant and complementation studies, we discovered that sX13 promotes the expression of the T3S system and contributes to virulence of *Xcv*. Microarray and quantitative reverse transcription PCR (qRT-PCR) analyses identified a large sX13 regulon and G-rich motifs in presumed sX13-target mRNAs. Selected putative targets were analyzed by site-directed mutagenesis of *sX13* and mRNA::*gfp* fusions. Furthermore, we provide evidence that sX13 acts Hfq-independently. Our study provides the first comprehensive characterization of a *trans*-encoded sRNA that contributes to virulence of a plant-pathogenic bacterium.

## Results

### *sX13* contributes to bacterial virulence

The sRNA sX13 (115 nt; [16]) is encoded in a 437-bp intergenic region of the *Xcv* 85-10 chromosome, i. e., 175 bp downstream of the stop codon of the DNA polymerase I-encoding gene *polA* and 148 bp upstream of the translation start site (TLS) of *XCV4199*, which encodes a hypothetical protein. Sequence comparisons revealed that the *sX13* gene is exclusively found in members of the *Xanthomonadaceae* family, i. e., in the genomes of plant-pathogenic *Xanthomonas* and *Xylella* species, the human pathogen *Stenotrophomonas maltophilia* and non-pathogenic bacteria of the genus *Pseudoxanthomonas*. Interestingly, *sX13* homologs are highly conserved [16] and always located downstream of *polA*. By contrast, *sX13*-flanking sequences are highly variable.

**Figure 1:**
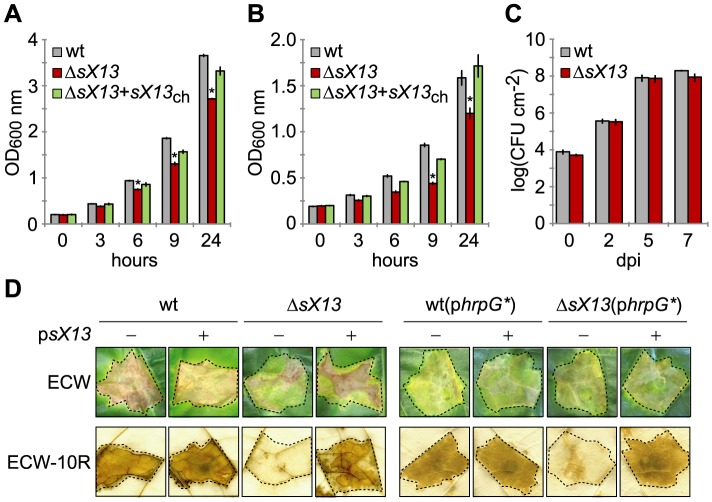
*sX13* contributes to bacterial growth in culture and virulence. Growth of *Xcv* wild type 85-10 (wt), the *sX13* deletion mutant (Δ*sX13*) and Δ*sX13* containing chromosomally re-integrated *sX13* (Δ*sX13*+*sX13*_ch_) in (A) complex medium NYG and (B) minimal medium MMA, respectively. Error bars represent standard deviations. Asterisks indicate statistically significant differences compared to wt (*t*-test; *P*<0.05). (C) Growth of *Xcv* 85-10 (wt) and Δ*sX13* in leaves of susceptible ECW pepper plants. Data points represent the mean of three different samples from three different plants of one experiment. Standard deviations are indicated by error bars. (D) Plant infection assay. *Xcv* strains 85-10 (wt) and Δ*sX13* carrying the empty vector (pB) or the sX13 expression construct (p*sX13*) and strains additionally expressing HrpG* (phrpG*) were inoculated at a density of 4×10^8^ (left panel) and 10^8^ cfu/ml (right panel), respectively, into leaves of susceptible ECW and resistant ECW-10R pepper plants. Disease symptoms in ECW were photographed 9 days post inoculation (dpi). The HR was visualized by ethanol bleaching of the leaves 3 dpi (left panel) and 18 hours post inoculation (right panel), respectively. Dashed lines indicate the inoculated areas. All experiments were performed at least three times with similar results.

To characterize *sX13* in *Xcv* strain 85-10, we introduced an unmarked *sX13* deletion into the chromosome (see ‘Materials and Methods’). Analysis of bacterial growth of the *sX13* mutant strain (Δ*sX13*) revealed a significantly reduced stationary-phase density compared to the *Xcv* wild-type strain 85-10 in complex medium (NYG; Figure 1A) and in minimal medium A (MMA; Figure 1B). The mutant phenotype of *Xcv*Δ*sX13* was complemented by chromosomal re-integration of *sX13* into the *sX13* locus, termed Δ*sX13*+*sX13*_ch_ (Figure 1A, B; see ‘Materials and Methods’).

To address a potential role of *sX13* in virulence, we performed plant infection assays. As shown in Figure 1C, the *Xcv* strains 85-10 and Δ*sX13* grew similarly in leaves of susceptible pepper plants (ECW). Strikingly, infection with the *sX13* mutant resulted in strongly delayed disease symptoms in susceptible and a delayed HR in resistant pepper plants (ECW-10R) (Figure 1D). Ectopic expression of sX13 under control of the *lac* promoter (p*sX13*), which is constitutive in *Xcv* [38], and re-integration of *sX13* into the Δ*sX13* locus fully complemented the mutant phenotype of *XcvΔsX13* (Figure 1D; data not shown). Strain *Xcv* 85-10 carrying p*sX13* induced an accelerated HR in comparison to the wild type (data not shown).

### Deletion of *sX13* derogates *hrp*-gene expression

**Figure 2:**
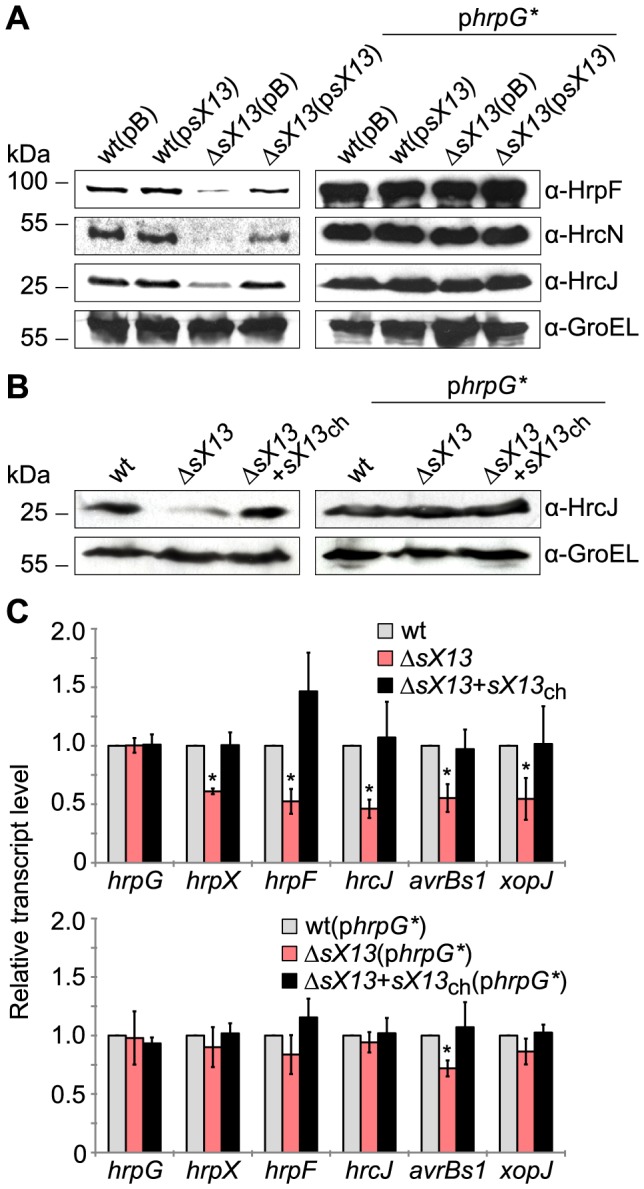
Deletion of *sX13* derogates virulence gene expression. (A) *Xcv* strains 85-10 (wt) and the *sX13* deletion mutant (Δ*sX13*) carrying the empty vector (pB) or the sX13 expression construct (p*sX13*) and strains additionally expressing HrpG* (phrpG*) were incubated for 3.5 hours in *hrp*-gene inducing medium XVM2. Total protein extracts were analyzed by immunoblotting using antibodies directed against HrpF, HrcN, HrcJ and GroEL. The experiment was repeated twice with similar results. (B) *Xcv* 85-10 (wt), Δ*sX13* and Δ*sX13*+*sX13*_ch_ and strains additionally expressing HrpG* were incubated for 3.5 hours in *hrp*-gene inducing medium XVM2. Total protein extracts were analyzed by immunoblotting using antibodies directed against HrcJ and GroEL. The experiment was repeated twice with similar results. (C) Indicated genes were analyzed by qRT-PCR using RNA from cultures described in (B). The amount of each RNA in *Xcv* 85-10 was set to 1. Data points and error bars represent mean values and standard deviations obtained with three independent biological samples. Asterisks indicate statistically significant differences compared to wt (*t*-test; *P*<0.03).

As the HR induction in ECW-10R plants depends on the recognition of the bacterial type III effector protein AvrBs1 by the plant disease resistance gene *Bs1* [44], [45], the *in planta* phenotype of *XcvΔsX13* suggested a reduced activity of the T3S system. To address this question, we investigated protein accumulation of selected components of the T3S system. Given that T3S apparatus proteins are not detectable in NYG-grown bacteria, we incubated the bacteria for 3.5 hours in the *hrp*-gene inducing XVM2 medium [38], [40]. Western blot analysis revealed reduced amounts of the translocon protein HrpF, the T3S-ATPase HrcN and the T3S-apparatus component HrcJ in *XcvΔsX13* compared to the wild type, Δ*sX13*(p*sX13*) (Figure 2A) and strain Δ*sX13*+*sX13*_ch_ (selectively tested for HrcJ; Figure 2B). Thus, sX13 positively affects the synthesis of T3S components.

As HrpG controls the expression of the *hrp*-regulon [39], we analyzed whether the reduced virulence of strain Δ*sX13* is due to a reduced activity of HrpG. Therefore, we ectopically expressed a constitutively active version of HrpG (HrpG*; phrpG*; [41]) in *Xcv*Δ*sX13* and performed plant-infection assays. The disease symptoms induced by *Xcv*Δ*sX13* and the wild type were comparable in presence of phrpG*, whereas with low inoculum of *Xcv* 85-10Δ*sX13* the HR was slightly delayed (Figure 1D). This suggests that HrpG* suppresses the 85-10Δ*sX13* phenotype. HrpF, HrcN and HrcJ protein accumulation in strain Δ*sX13*(phrpG*) was identical to the wild type suggesting full complementation (Figure 2A, B).

To investigate whether the reduced protein amounts of T3S-system components in *Xcv*Δ*sX13* are due to altered mRNA levels, we performed qRT-PCR analyses. mRNA accumulation of *hrpF*, *hrcJ* and the type III effector genes *avrBs1* and *xopJ* was two-fold lower in *Xcv*Δ*sX13* than in the wild type and the complemented strain Δ*sX13*+*sX13*_ch_ (Figure 2C). In addition, the mRNA amount of *hrpX*, but not of *hrpG*, was reduced in the *sX13* mutant (Figure 2C). In presence of phrpG*, comparable mRNA amounts of *hrpG*, *hrpX*, *hrpF*, *hrcJ* and *xopJ* were detected in *Xcv* 85-10, Δ*sX13* and Δ*sX13*+*sX13*_ch_, whereas the *avrBs1* mRNA accumulation was significantly reduced in strain 85-10Δ*sX13* (Figure 2C). Taken together, our data suggest that the reduced virulence of the 85-10Δ*sX13* mutant is caused by a lower expression of components and substrates of the T3S system (Figure 1D; Figure 2A–C).

The deletion and chromosomal re-insertion of *sX13* in *Xcv*Δ*sX13* and Δ*sX13*+*sX13*_ch_, respectively, were verified by Northern blot using an sX13-specific probe (Figure S1). The sX13 abundance was not affected by expression of HrpG*, which confirms our previous findings [16] and suggests that expression of sX13 is independent of HrpG and HrpX (Figure S1).

### sX13 accumulates under stress conditions

**Figure 3:**
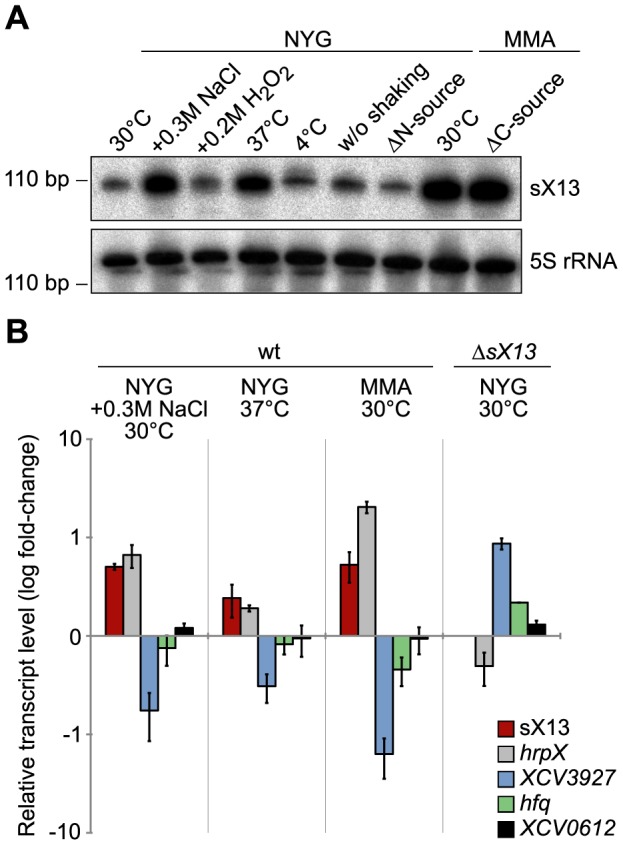
sX13 accumulation is altered under stress conditions in *Xcv* 85-10. (A) Northern blot analysis of sX13. Exponential phase cultures of NYG-grown *Xcv* 85-10 were transferred to NYG medium or MMA containing the indicated additives or lacking a nitrogen or carbon source (ΔN; ΔC). Cultures were shaken for three hours at 30°C unless otherwise indicated. 5S rRNA was probed as loading control. (B) sX13 and selected sX13-regulated genes (see Table 1) were analyzed by qRT-PCR using RNA from *Xcv* 85-10 (wt) cultures shown in (A) and NYG-grown Δ*sX13*. Bars represent fold-changes (log_10_) of mRNA amounts compared to *Xcv* 85-10 grown in NYG at 30°C. Experiments were performed twice with similar results.

The expression of known bacterial sRNAs depends on a variety of environmental stimuli, which often reflect the physiological functions of sRNAs [2], [46], e. g., the *E. coli* sRNA Spot42 is repressed in the absence of glucose and regulates carbon metabolism [47], [48]. Northern blots revealed similar sX13 amounts in bacteria incubated in NYG medium at 30°C (standard condition), in presence of H_2_O_2_, at 4°C and in NYG medium lacking nitrogen (Figure 3A). By contrast, sX13 accumulation was increased in presence of high salt (NaCl), 37°C and in MMA (Figure 3A). Hence, sX13 is differentially expressed in different growth conditions and might contribute to environmental adaptation of *Xcv*.

### Microarray analyses suggest a large sX13 regulon

**Figure 4:**
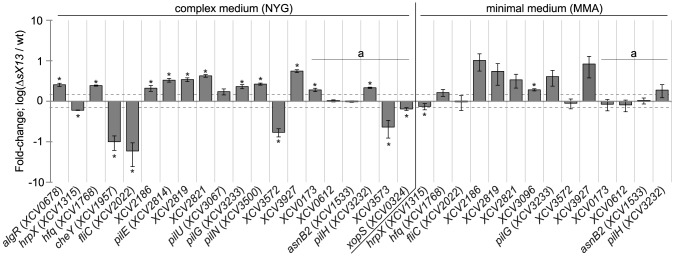
qRT-PCR analysis of sX13-regulated genes. Selected sX13-regulated genes (see Table 1) were analyzed by qRT-PCR using RNA from NYG- and MMA-grown *Xcv* strains 85-10 (wt) and Δ*sX13*. The amount of each mRNA in the wt was set to 1. Bars represent fold-changes of mRNA amounts in strain Δ*sX13* compared to 85-10 on a logarithmic scale (log_10_). Data points and error bars represent mean values and standard deviations obtained with at least three independent biological samples. Asterisks denote statistically significant differences (*t*-test; *P*<0.05). Dashed lines indicate a 1.5-fold change. Transcripts not detected in the microarray analyses are marked with ‘a’.

To gain an insight into the sX13 regulon we performed microarray analyses. For this, cDNA derived from *Xcv* strains 85-10 and Δ*sX13* grown in NYG and MMA, respectively, was used as a probe. The hybridization data were evaluated using EMMA 2.8.2 [49] (see ‘Materials and Methods’). In *XcvΔsX13* grown in NYG, 23 mRNAs were upregulated and 21 mRNAs were downregulated by a factor of at least 1.5 compared to the wild type (Table S2). In the MMA-grown *sX13* mutant, 23 upregulated mRNAs were detected, four of which were also upregulated in NYG-grown bacteria, whereas no downregulated genes were identified (Table S2). With respect to both growth conditions, 42 and 21 genes were upregulated and downregulated, respectively, in the *sX13* mutant. qRT-PCR analyses of 11 selected upregulated and four downregulated genes confirmed the microarray data (Table 1; Figure 4).

**Table 1:** Selected sX13-regulated genes validated by qRT-PCR analysis.

Locus^a^	Annotated gene product^b^	4G-motif^c^	Microarray – Fold-change (Δ*sX13*/wt)^d^		qRT-PCR – Fold-change (Δ*sX13*/wt)^e^	
			NYG	MMA	NYG	MMA
**Upregulated genes (Δ** ***sX13*** **/wt)**						
XCV0678	AlgR; two-component system regulatory protein	a,a,a	1.8	—	2.5±0.23	n.t.
**XCV1768**	Hfq; host factor-I protein	b	1.6	—	2.4±0.08	1.6±0.31
XCV2186	methyl-accepting chemotaxis protein	a	7.7	—	2.1±0.34	10.2±4.63
XCV2814	PilE; type IV pilus pilin	—	2.8	—	3.3±0.36	n.t.
XCV2819	type IV pilus assembly protein PilW	a	3.7	4.0	3.4±0.37	5.5±3.0
**XCV2821**	type IV pilus assembly protein FimT	a	4.3	7.4	4.2±0.32	3.4±1.27
XCV3067	PilU; type IV pilus assembly protein ATPase	a	1.8	—	1.7±0.29	n.t.
**XCV3096**	ComEA-related DNA uptake protein	—	—	4.2	n.t.	1.9±0.12
**XCV3233**	PilG; type IV pilus response regulator	a,b	—	2.0	2.3±0.26	4.1±1.71
**XCV3500**	PilN; type IV pilus assembly protein	—	2.7	—	2.7±0.16	n.t.
**XCV3927**	putative secreted protein	a	—	1.7	5.6±0.45	8.3±4.54
**Downregulated genes (Δ** ***sX13*** **/wt)**						
XCV1315	HrpX; AraC-type transcriptional regulator	—	0.6	—	0.6±0.01	0.7±0.13
XCV1957	CheY; chemotaxis response regulator	—	0.4	—	0.1±0.04	n.t.
**XCV2022**	FliC; flagellin and related hook-associated proteins	—	0.2	—	0.06±0.03	1.0±0.39
XCV3572	TonB-dependent outer membrane receptor	a	0.2	—	0.2±0.04	0.9±0.24
**Additional genes tested by qRT-PCR**						
XCV0173	putative secreted protein	a,b,b,b	—	—	1.9±0.19	0.8±0.26
**XCV0612**	ATPase of the AAA+ class	a	—	—	1.0±0.06	0.8±0.26
XCV1533	AsnB2; asparagine synthase	b	—	—	1.0±0.04	1.0±0.17
XCV3232	PilH; type IV pilus response regulator	a	—	—	2.2±0.07	1.9±0.67
XCV3573	putative transcriptional regulator, AraC family	a	—	—	0.2±0.11	n.t.
XCV0324	type III effector protein XopS	—	—	—	0.6±0.05	n.t.

a , bold letters indicate genes with known TSS [16].

b , refers to Thieme *et al.* (2005) [32].

c , presence of a 4G-motif within the 5′-UTR or 100 bp upstream of translation start codon if TSS is unknown (a) and within 100 bp downstream of start codon (b) (see Figure S4).

d , genes not detected as expressed are marked with —.

e , values represent mean fold-change and standard deviation (see Figure 4);

n.t. - not tested.

### sX13 negatively affects *hfq* and type IV pilus-biosynthesis mRNAs

Based on the annotated genome sequence of *Xcv* 85-10 [32], genes upregulated in *XcvΔsX13* can be grouped (Table S2): 18 genes encode proteins with unknown function, e. g., the putative LysM-domain protein XCV3927. 14 genes encode proteins involved in type IV pilus (Tfp) biogenesis, e. g., the putative Tfp assembly protein XCV2821, the pilus component PilE and the TCS response regulator PilG. Tfp enable twitching motility, i. e., adhesion to and movement on solid surfaces [50], [51]. Three genes encode proteins assigned to signal transduction, i. e., the TCS regulator AlgR, the GGDEF-domain protein XCV2041 and the chemotaxis regulator XCV2186. Moreover, *hfq* mRNA accumulation was two-fold increased in *XcvΔsX13*.

The microarray data suggested that most upregulated genes in *Xcv*Δ*sX13* were only expressed in NYG- or MMA-grown bacteria (Table S2), which might be explained by the *P*-value and signal-strength thresholds applied for data evaluation. qRT-PCR analyses showed that the mRNA accumulation of *hfq*, *XCV2186*, *pilG* and *XCV3927* was increased in both the NYG- and MMA-grown *sX13* mutant compared to the wild type (Figure 4; Table 1). qRT-PCR analyses also revealed an upregulation of *pilH* in the NYG- and MMA-grown *Xcv*Δ*sX13* compared to the wild type (Figure 4; Table 1). Because *pilH* is the second gene in the *pilG* operon and was not detected as expressed in the microarray data, the number of mRNAs affected by *sX13* deletion might be higher than suggested by the microarray data.

### sX13 positively affects *hrpX* and chemotaxis-regulating mRNAs

Five of 21 genes downregulated in *Xcv*Δ*sX13* presumably encode proteins involved in flagellum-mediated chemotaxis, e. g., the sensor kinase CheA1, the corresponding response regulator CheY and the flagellum components FliD and FliC (Table S2). qRT-PCR analyses revealed 17-fold lower *fliC* mRNA abundance in *Xcv*Δ*sX13* grown in NYG compared to the wild type, whereas the accumulation in MMA-grown cells was identical (Figure 4; Table 1). Similarly, *XCV3572*, which encodes a TonB-dependent receptor, was downregulated in NYG- but not in MMA-grown *Xcv*Δ*sX13* (Figure 4; Table 1). Gene *XCV3573*, which is encoded adjacent to *XCV3572* and encodes an AraC-type regulator, was also downregulated (Figure 4; Table 1). As mentioned above, sX13 positively affected the mRNA accumulation of *hrpX* in XVM2 medium (see Figure 2C), which was also true for bacteria grown in NYG and MMA (Figure 4; Table 1). Since HrpX controls the expression of many type III effector genes, we analyzed *xopS* [52] by qRT-PCR and detected similarly decreased levels in NYG-grown *Xcv*Δ*sX13* as for *hrpX* (Figure 4; Table 1). Taken together, our data suggest that the sX13 regulon comprises genes involved in signal transduction, motility, transcriptional and posttranscriptional regulation and virulence.

### Accumulation of potential target mRNAs correlates with sX13 abundance

To address whether differential expression of sX13 under different conditions (see Figure 3A) affects the mRNA abundance of sX13-regulated genes, we performed qRT-PCR. We detected elevated sX13 levels in *Xcv* strain 85-10 cultivated in high salt conditions, at 37°C and in MMA compared to standard conditions and an increased *hrpX* and decreased *XCV3927* mRNA accumulation (Figure 3B). In addition, low amounts of the *hfq* mRNA were detected in presence of high sX13 levels, whereas the abundance of the sX13-independent *XCV0612* mRNA (see Table 1) was not altered (Figure 3B).

### sX13 activity does not require Hfq

**Figure 5:**
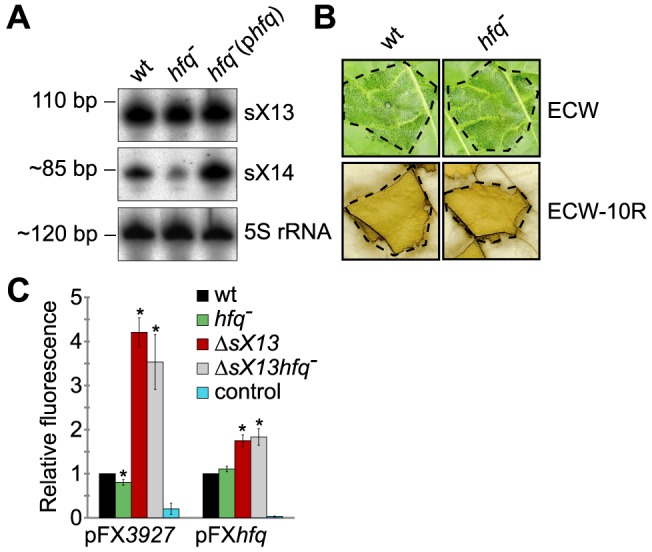
sX13 activity does not require Hfq. (A) Northern blot analysis. Total RNA from NYG-grown *Xcv* strains 85-10 (wt), the *hfq* frameshift mutant (*hfq^−^*) and the *hfq* mutant ectopically expressing Hfq (p*hfq*) was analyzed using sX13- or sX14-specific probes. 5S rRNA was probed as loading control. The experiment was performed twice with two independent mutants and with similar results. (B) Plant infection assay. The *Xcv* wild-type 85-10 (wt) and *hfq* mutant strain (*hfq^−^*) were inoculated at 2×10^8^ cfu/ml into leaves of susceptible ECW and resistant ECW-10R plants. Disease symptoms were photographed 6 dpi. The HR was visualized 2 dpi by ethanol bleaching of the leaves. Dashed lines indicate the inoculated areas. The experiment was repeated three times with similar results. (C) GFP fluorescence of NYG-grown *Xcv* 85-10 (wt), the *hfq* mutant (*hfq^−^*), the *sX13* deletion mutant (Δ*sX13*) and the double mutant (Δ*sX13hfq^−^*) carrying pFX*3927* or pFX*hfq*. *Xcv* autofluorescence was determined by *Xcv* 85-10 carrying pFX0 (control). Data points and error bars represent mean values and standard deviations obtained with at least four independent experiments. GFP fluorescence of the wt was set to 1. Asterisks denote statistically significant differences (*t*-test; *P*<0.01).

The *hfq* mRNA accumulation in *Xcv*Δ*sX13* (Figure 3B; Figure 4; Table 1) prompted us to test whether sX13 activity depends on the RNA-binding protein Hfq. For this, we introduced a frameshift mutation into the *hfq* gene of *Xcv* strains 85-10 and 85-10Δ*sX13*. Northern blot analyses revealed comparable sX13 accumulation in both strains and the complemented *hfq* mutant, which ectopically expressed Hfq (p*hfq*) (Figure 5A). By contrast, the accumulation of the sRNA sX14 [16] was strongly reduced in the *hfq* mutant; this was restored by p*hfq* (Figure 5A). Unexpectedly, the *hfq* mutant strain was not altered in the induction of *in planta* phenotypes, i. e., in virulence (Figure 5B).

To investigate whether sX13 affects translation of putative target mRNAs, we established a GFP-based *in vivo* reporter system for *Xcv* similar to the one described for *E. coli* [53]. The promoterless broad-host range plasmid pFX-P permits generation of translational *gfp* fusions in a one-step restriction-ligation reaction (Golden Gate cloning [54]; see ‘Materials and Methods’). We cloned the promoter, 5′-UTRs, and 10 and 25 codons of *XCV3927* and *hfq*, respectively, into pFX-P resulting in pFX*3927* and pFX*hfq*. *XCV3927* was selected because of a strongly increased mRNA accumulation in *XcvΔsX13* compared to the wild type (see Table 1). In presence of pFX*3927* or pFX*hfq*, fluorescence of XCV3927::GFP or Hfq::GFP fusion proteins was comparable in the *Xcv* wild type and *hfq* mutant (Figure 5C). The XCV3927::GFP and Hfq::GFP fluorescence was about 4-fold and 2-fold increased, respectively, in *XcvΔsX13* compared to strain 85-10 (Figure 5C), suggesting that the synthesis of the fusion proteins is repressed by sX13. Interestingly, the XCV3927::GFP and Hfq::GFP fluorescence was similarly increased in *XcvΔsX13* and the *sX13hfq* double mutant (Figure 5C). As abundance and activity of sX13 were not affected by the *hfq* mutation, we assume that sX13 acts Hfq-independently.

### sX13 activity *in planta* depends on C-rich loop motifs

**Figure 6:**
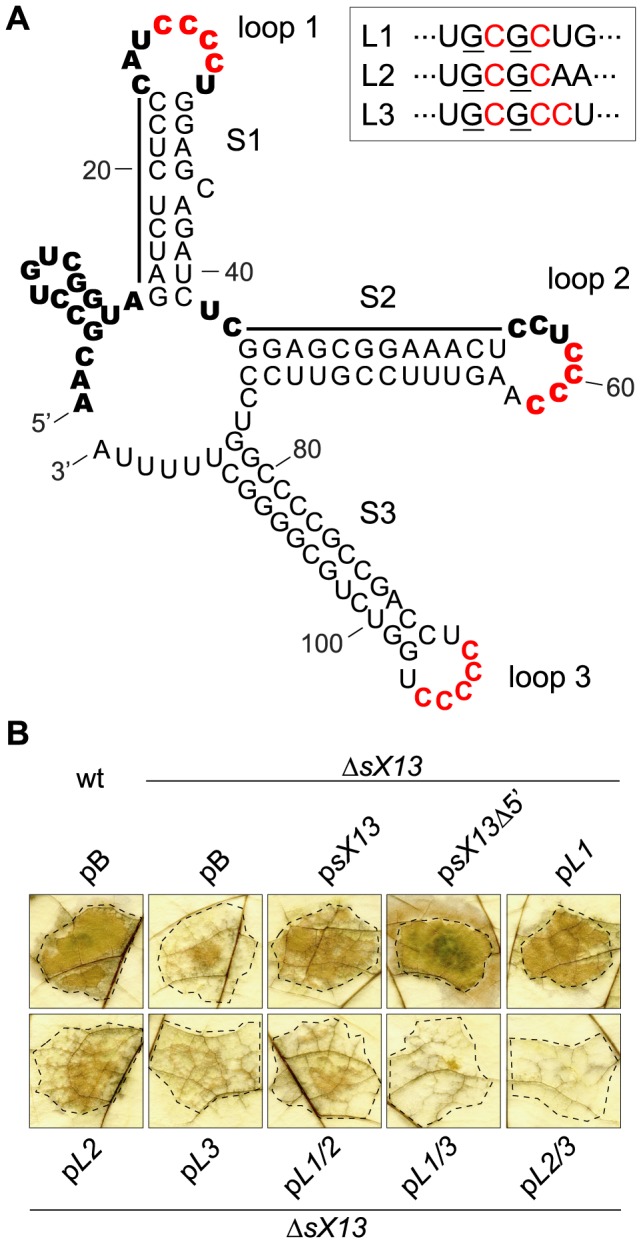
sX13 loops impact on *Xcv* virulence. (A) Secondary structure of sX13 based on prediction and probing (see Figure S2). sX13 consists of an unstructured 5′-, three double-stranded regions (S1; S2; S3) and three loops (loop 1–3). 4C-/5C-motifs are highlighted in red. Bold letters indicate unpaired bases and bars mark double-stranded regions deduced from structure probing. Mutations in loops are boxed, exchanged nucleotides are underlined. (B) Derivatives mutated in loops 2 and 3 fail to complement the plant phenotype of Δ*sX13*. Leaves of resistant ECW-10R plants were inoculated at 10^8^ cfu/ml with *Xcv* 85-10 (wt) and Δ*sX13* carrying pBRS (pB), p*sX13* or one of the following derivatives: sX13 lacking 14 terminal nucleotides (p*sX13*Δ5′), sX13 mutated in single loops (p*L1*, p*L2*, p*L3*) or in two loops (p*L1/2*, p*L1/3*, p*L2/3*). The HR was visualized by ethanol bleaching of the leaves 2 dpi. Dashed lines indicate the inoculated areas. The experiment was performed four times with similar results.

The predicted secondary structure of sX13 obtained by mfold [55] displays an unstructured 5′-region and three stable stem-loops, termed stem 1 to 3, and loop 1 to 3 (Figure 6A). Interestingly, loop 1 and loop 2 contain a ‘CCCC’ (4C) motif, whereas loop 3 harbors a ‘CCCCC’ (5C) motif (Figure 6A). To experimentally verify the predicted structure, we performed structure analyses of *in vitro* transcribed and radioactively-labeled sX13 treated with RNase V1 or RNase T1. While RNase T1 cleaves single-stranded RNA with a preference for G residues, RNase V1 randomly cleaves double-stranded RNA. We detected RNase T1-cleavage products for the 5′-region and RNase V1-cleavage products for stem 1 and 2, which is in good agreement with the predicted structure (Figure 6A; Figure S2). Moreover, RNase V1-cleavage products were less abundant for the 4C-motif of loop 1 and loop 2, suggesting single-stranded sequences (Figure 6A; Figure S2). The results did not allow conclusions about stem 3 and loop 3 structures.

To assess the contribution of the 4C-/5C-motifs to sX13 function, we mutated p*sX13* in loop 1 and 2, respectively, to ‘GCGC’, and the 5C-motif in loop 3 to ‘GCGCC’ resulting in p*L1*, p*L2* and p*L3* (Figure 6A). In addition, loop mutations were combined (p*L1/2*, p*L1/3*, p*L2/3*) and analyzed for their ability to complement the *in planta* phenotype of strain Δ*sX13*. As shown above, *Xcv*Δ*sX13* induced a delayed HR, which was complemented by p*sX13* (Figure 1D). Similar phenotypes were observed with *sX13* mutants carrying p*L1* or p*sX13Δ5*′, which encodes a 5′-truncated sX13 derivative lacking the terminal 14 nucleotides (Figure 6B). The HR induced by the *sX13* mutant containing p*L2* or p*L1/2* was intermediate, whereas p*L3*, p*L1/3* and p*L2/3* failed to complement *XcvΔsX13* (Figure 6B). Northern blot analyses revealed expression of all *sX13*-loop mutant derivatives (Figure S3). The different RNA species derived from ectopically expressed sX13 and derivatives compared to chromosomally encoded sX13 might be due to alternative transcription termination of plasmid-derived sX13 and derivatives.

### sX13 loops differentially contribute to mRNA accumulation

**Figure 7:**
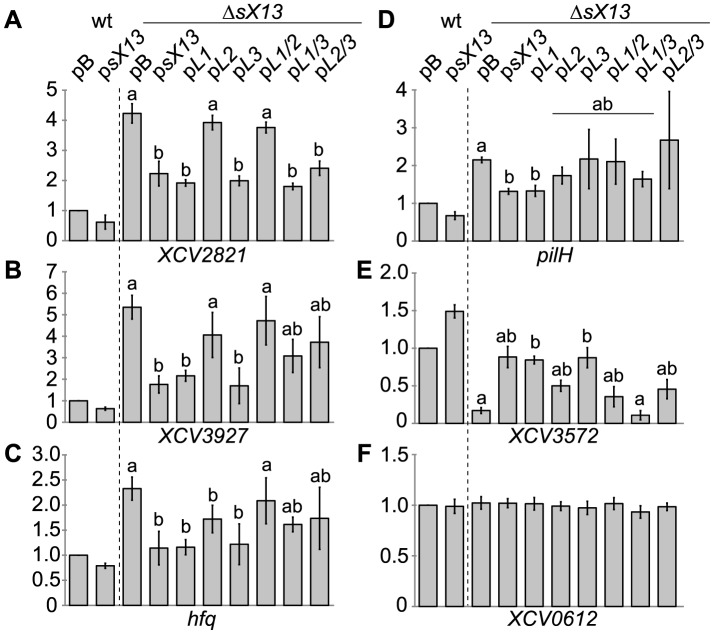
sX13 loops differentially contribute to abundance of putative mRNA targets. Relative transcript levels of (A) *XCV2821*, (B) *XCV3927*, (C) *hfq*, (D) *pilH*, (E) *XCV3572* and (F) *XCV0612* were analyzed by qRT-PCR in total RNA of NYG-grown *Xcv* strains 85-10 (wt) and Δ*sX13* carrying pBRS (pB), p*sX13* or mutated sX13-derivatives (see Figure 6). The mRNA abundance in the wt was set to 1. Data points and error bars represent mean values and standard deviations obtained with at least three independent biological samples. Statistically significant differences are indicated (*t*-test; *P*<0.015).

As mutation of sX13 loops impinged on *Xcv* virulence (Figure 6B), we addressed by qRT-PCR whether loop mutations affect the mRNA abundance of *XCV2821*, *XCV3927*, *hfq* and *pilH*, which were upregulated in *XcvΔsX13* (see Figure 4; Table 1). In addition, we analyzed a downregulated gene, *XCV3572*, and *XCV0612*, which was not affected by *sX13* deletion. As shown in Figure 7A–E, sX13 negatively affected the mRNA abundance of *XCV2821*, *XCV3927*, *hfq* and *pilH*, whereas sX13 promoted mRNA accumulation of *XCV3572*. Mutation of sX13 loops differentially affected the mRNA abundance of the tested genes: p*L2* and p*L1/2* failed to complement *Xcv*Δ*sX13* with respect to the mRNA abundance of *XCV2821*, *XCV3927* and *hfq* (Figure 7A–C). Intermediate mRNA amounts of *XCV3927* and *hfq* were detected in *XcvΔsX13* carrying p*L1*/*3* or p*L2*/*3* compared to pB and p*sX13* (Figure 7B, C). Taken together, the mRNA abundance of *XCV2821*, *XCV3927* and *hfq* appears to be mainly controlled by sX13-loop 2. In contrast, *pilH* mRNA accumulation appears to depend on multiple sX13 loops as only p*sX13* and p*L1* complemented *XcvΔsX13* (Figure 7D). The reduced mRNA amount of *XCV3572* in *XcvΔsX13* was complemented by p*L1* and p*L3* but not by p*L1*/3 (Figure 7E), which suggests redundant roles of sX13-loops. In presence of p*L2*, p*L1*/*2* or p*L2*/*3* in *XcvΔsX13*, the *XCV3572* mRNA levels were intermediate compared to *XcvΔsX13* carrying pB or p*sX13* (Figure 7E). As expected, the mRNA abundance of *XCV0612* was identical in the different strains (Figure 7F).

### Identification of putative sX13-binding sites

To identify potential regulatory motifs in sX13-regulated mRNAs, a discriminative motif search was performed using DREME [56]. For this, sequences surrounding the TLSs of the 42 up- and 21 downregulated genes identified by microarray analyses (Table S2) were compared. More precisely, sequences spanning from known transcription start sites (TSSs) to 100 bp downstream of TLSs or, in case of unknown TSSs, 100 bp up- and 100 bp downstream of the TLS were inspected.

We found that up- and downregulated genes differ in the presence of ‘GGGG’ (4G) motifs. In the NYG-grown *sX13* mutant, 15 out of 23 (65%) upregulated genes contain up to three 4G-motifs which are predominantly located upstream of the TLS (Figure S4A; Table S2). 70% of the genes upregulated in MMA (16 out of 23), but only 14% of the genes downregulated in NYG medium (3 out of 21) contain 4G-motifs (Figure S4A; Table S2). Thus, 4G-motifs appear to be enriched in sX13-repressed mRNAs. However, the position of the motifs and flanking nucleotides are not conserved among sX13-regulated genes. Note that the term ‘4G-motif’ also refers to motifs containing more than four G-residues in a row. The complementarity of C-rich sX13-loop sequences and G-rich mRNA motifs suggests sX13-mRNA interactions via antisense-base pairing (Figure 6A; Table 1; Table S2).

Compared to the occurrence of 4G-motifs in approximately 70% of sX13-repressed genes, only 30.71% of all chromosomally encoded *Xcv* genes (1,378 out of 4,487) carry 4G-motifs in proximity of their TLS (Figure S4A). Interestingly, 4G-motifs in 241 of the chromosomally encoded genes (5.37%) are located between nucleotide position 8 and 15 upstream of the TLS (Figure S4B). This position corresponds to the presumed location of the RBS and suggests a role of 4G-motifs in translation control.

### sX13 dependency of target::GFP synthesis requires both 4C- and 4G-motifs

**Figure 8:**
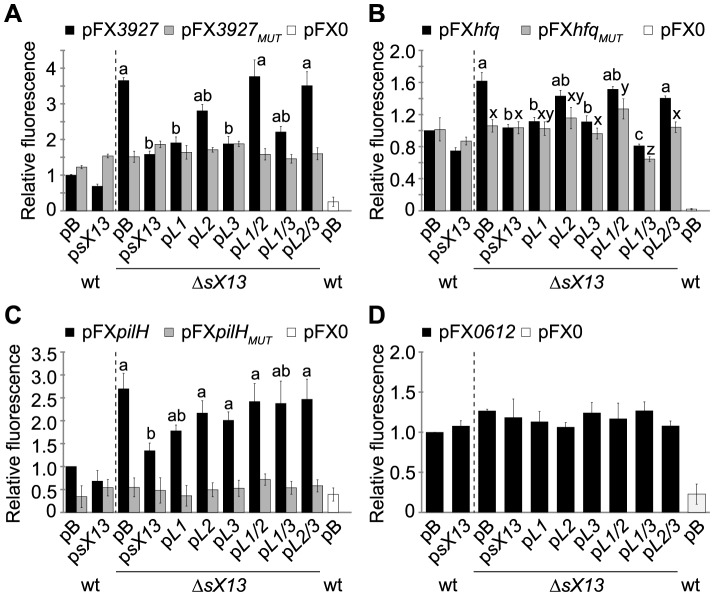
sX13-dependency of mRNA target::GFP synthesis requires a G-rich motif. GFP fluorescence of NYG-grown *Xcv* strains 85-10 (wt) and Δ*sX13* carrying pB, p*sX13* or mutated sX13-derivatives (see Figure 6) and carrying GFP-reporter plasmids (A) pFX*3927*, (B) pFX*hfq*, (C) pFX*pilH* or (D) pFX*0612*. pFX*_MUT_* derivatives contain a mutated 4G-motif. *Xcv* autofluorescence was determined using pFX0. GFP fluorescence of the wt was set to 1. Data points and error bars represent mean values and standard deviations obtained from at least four independent experiments. Statistically significant differences are indicated (*t*-test; *P*<0.015).

To study the effect of sX13 on translation of selected putative targets, i. e., *XCV3927* and *hfq*, we used the above-mentioned GFP-reporter plasmids pFX*3927* and pFX*hfq*. In addition, we generated *pilH::gfp* (pFX*pilH*) and *XCV0612::gfp* (pFX*0612*) fusions (see ‘Materials and Methods’). All mRNA::*gfp* fusions contain a G-rich motif in the proximity of their TLS which is complementary to C-rich sX13-loop regions (see ‘Materials and Methods’). The fluorescence of the *sX13* deletion mutant carrying pFX*3927*, pFX*hfq* and pFX*pilH* was about 3.5-, 1.6- and 2.5-fold higher, respectively, compared to the *Xcv* wild type (Figure 8A–C). In presence of p*sX13*, p*L1*, p*L3* or p*L1*/*3* in *XcvΔsX13*, the XCV3927::GFP and Hfq::GFP fluorescence levels were comparable to the *Xcv* wild type (Figure 8A, B). By contrast, the XCV3927::GFP and Hfq::GFP fluorescence of strain Δ*sX13* carrying p*L2*, p*L1*/*2* or p*L2/3* was similarly increased as compared to *Xcv*Δ*sX13* carrying pB (Figure 8A, B). This suggests that the 4C-motif in sX13-loop 2 is required to repress XCV3927::GFP and Hfq::GFP synthesis. The increased PilH::GFP fluorescence of *XcvΔsX13* was complemented by p*sX13* and p*L1*, in contrast to other *sX13*-loop mutant derivatives (Figure 8C). Fluorescence values of all analyzed *Xcv* strains carrying pFX*0612* were comparable confirming *sX13*-independency (Figure 8D).

To address whether the G-rich motif in presumed target mRNAs is required for sX13 dependency of mRNA::*gfp* translation, we introduced compensatory mutations. i. e., mutated the motif to ‘GCGC’. *Xcv* strains carrying the resulting plasmids, pFX*3927_MUT_*, pFX*hfq_MUT_* or pFX*pilH_MUT_*, exhibited a similar fluorescence in absence and presence of sX13 and mutated sX13 derivatives (Figure 8A–C). This suggests that the G-rich motif is required for sX13-dependency of target::GFP synthesis. However, mutation of the C-rich motifs in sX13 and the G-rich motifs in mRNA::*gfp* fusions did not restore sX13 dependency (Figure 8A–C). Unexpectedly, the fluorescence detected for *Xcv* strains containing pFX*3927_MUT_* or pFX*hfq_MUT_* was comparable to the fluorescence of *Xcv* 85-10 carrying the non-mutated plasmids pFX*3927* and pFX*hfq*, respectively (Figure 8A, B). The mutation of the 5G-motif in *pilH* abolished the fluorescence of strains containing pFX*pilH_MUT_* indicating an essential role of the motif in *pilH* translation (Figure 8C).

Because sX13 was more abundant in MMA- than NYG-grown bacteria (Figure 3), we also analyzed the fluorescence of MMA-grown *Xcv* strains containing pFX-derivatives. XCV3927::GFP and PilH::GFP synthesis in MMA was sX13-dependently repressed to a greater extent than in NYG (Figure S5; see Figure 8).

Because sX13 negatively affected both the mRNA accumulation of chromosomally encoded *XCV3927*, *hfq* and *pilH* genes and accumulation of the corresponding GFP-fusion proteins, we exemplarily analyzed whether this is due to an altered mRNA abundance. However, qRT-PCR analyses revealed that the *XCV3927::gfp* mRNA accumulation was sX13-independent suggesting that sX13 posttranscriptionally affects the synthesis of XCV3927::GFP (Figure S6).

To discriminate between transcriptional and posttranscriptional effects of sX13 on target::GFP synthesis we generated reporter fusions controlled by p*lac* (see ‘Materials and Methods’). Note that the activity of the *lac* promoter is not affected by deletion of *sX13* (data not shown). As shown in Figure S7, the fluorescence of *XcvΔsX13* carrying pFXpl-*3927* (*XCV3927*) and pFXpl-*pilH* (*pilH*) was 2.5- and 4-fold higher, respectively, compared to the *Xcv* wild type and the complemented *sX13* mutant strain. Interestingly, mutation of the 4G-motif in the *XCV3927* 5′-UTR did not only abolish sX13-dependency but also led to a significantly reduced fluorescence compared to the *Xcv* wild type which carried the non-mutated reporter plasmid (Figure S7). This suggests that the 4G-motif in the *XCV3927* 5′-UTR promotes translation, i. e., acts as translational enhancer element. In presence of pFXpl-*pilH*, the fluorescence of the fusion protein was only detectable in the *sX13* mutant but not in the wild type or complemented strain, confirming that PilH::GFP synthesis is repressed by sX13 (Figure S7). Overall, the data confirm that sX13 represses the synthesis of XCV3927 and PilH on the posttranscriptional level.

## Discussion

### sX13 controls *Xcv* virulence

This study provides a first insight into the posttranscriptional modulation of clade-specific adaptive processes in a plant-pathogenic γ-proteobacterium. We identified sX13 as a major regulator of *Xcv* virulence in that it promotes expression of genes in the *hrp*-regulon, i. e., components and substrates of the T3S system (Figure 2A–C). This finding is remarkable because the *hrp*-regulon is only expressed under certain conditions, whereas sX13 is constitutively expressed (Figure S1) [16]. The *sX13* gene is exclusively found and highly conserved in members of the *Xanthomonadaceae* family of Gram-negative bacteria [16]. Intriguingly, several species with an *sX13* homolog lack a T3S system, e. g., the plant pathogen *X. fastidiosa* and the opportunistic human pathogen *S. maltophilia*. This suggests a role of sX13 apart from regulation of the *hrp*-regulon in these organisms.

The expression of the *hrp*-regulon depends on HrpG and HrpX [39], [40]. HrpG is presumably posttranslationally activated in the plant and in XVM2 medium and induces the expression of *hrpX* [38], [39], [40], [41]. As the XVM2-grown *sX13* mutant displayed decreased mRNA amounts of *hrpX* but not of *hrpG* (Figure 2C), we suppose that sX13 acts upstream of HrpG. This idea is supported by the finding that constitutively active HrpG (HrpG* [41]) suppressed the *sX13* mutation with respect to virulence and the expression of *hrpX* and downstream genes (Figure 1D; Figure 2A–C). In addition, sX13 affected the basal expression level and, hence, the activity of HrpX under non-inducing conditions, which might impact on the efficiency of *hrp*-gene induction during infection. Based on the fact that HrpG::GFP and HrpX::GFP synthesis was sX13-independent (Figure S8) we assume that sX13 indirectly controls the expression of the *hrp*-regulon.

### Physiological roles of sX13

Deletion of *sX13* affected the mRNA abundance of more than 60 genes involved in signaling, motility, transcriptional and posttranscriptional regulation (Table S2). sX13 negatively regulated mRNAs involved in Tfp biogenesis but promoted the accumulation of mRNAs involved in flagellum-mediated chemotaxis in a growth-phase dependent manner (Table 1; Table S2). This, together with the fact that sX13 is differentially expressed under certain stress conditions (Figure 3), implies a central role of sX13 in the transduction of environmental signals into comprehensive cellular responses affecting virulence gene expression, motility and QS regulation. The latter is corroborated by the reduced stationary-phase cell density of the *sX13* mutant compared to the *Xcv* wild type (Figure 1A, B) and the sX13-dependency of the *XCV2041* mRNA (Table S2), which encodes a GGDEF-/EAL-domain protein. Such domains play a role in the control of cyclic-di-GMP levels and QS regulation [57]. Interestingly, XCV2041 shares 94% identity with the *Xcc* protein XC2226 which is a repressor of Tfp-mediated motility [58].

Another remarkable finding of our study was the sX13-dependent accumulation of the *hfq* mRNA. To the best of our knowledge, sX13 is the first sRNA which affects expression of this conserved RNA-binding protein (Table 1). The *Xcv hfq* mutant was unaltered in virulence on its host plant (Figure 5B), which is in good agreement with recent findings for *Xoo* [18]. By contrast, Hfq contributes to virulence in a number of other bacteria, including the plant pathogen *A. tumefaciens*, and is also involved in symbiotic plant interactions of *Sinorhizobium meliloti* [5], [12], [59], [60]. In *Vibrio cholerae*, four redundantly acting and Hfq-dependent sRNAs (Qrr) destabilize *hapR* mRNA, which encodes the master regulator of QS, the T3S system and other virulence genes [61], [62]. In the Gram-positive human pathogen *Staphylococcus aureus*, the Hfq-independent RNAIII is induced by the *agr* QS system and mediates the switch between the expression of surface proteins and excreted toxins through translational repression of Rot (repressor of toxins) [63], [64], [65].

### sX13 activity depends on C-rich loop regions

*Xcv* sRNAs are strongly structured and lack extended single-stranded regions [15], [16], [32]. In contrast, enterobacterial sRNAs commonly exhibit a modular structure consisting of a single-stranded mRNA-targeting domain, often located at the 5′-end, an A/U-rich Hfq-binding site and a Rho-independent terminator [1]. The sX13 structure suggests that direct sRNA-mRNA interactions are energetically confined to the unstructured 5′-region and its three C-rich loops (Figure 6A). However, the 5′-region of sX13 was dispensable for full virulence of *Xcv* and sX13 activity appears to be exerted via loops 2 and 3 (Figure 6B). Although loops 1 and 2 just differ in the 3′-adjacent nucleotide (U/A) (Figure 6A), only loop 2 was required to repress the synthesis of XCV3927::GFP and Hfq::GFP, which might depend on the position of stem-loops in the sRNA and, thus, accessibility. By contrast, repression of PilH::GFP appears to depend on multiple sX13 regions (Figure 8C).

An important question is whether sX13 controls target gene expression on the level of mRNA stability or translation. On one hand, sX13-loop mutant derivatives affected the mRNA levels of presumed targets (Figure 7). On the other hand, protein levels, but not the mRNA level of an *XCV3927::gfp* fusion, harboring only the 5′-UTR and 10 codons of *XCV3927*, were sX13-dependent (Figure 8A; Figure S6). This suggests that the impact of sX13 on *XCV3927* mRNA abundance and translation are separate events and hints at the presence of additional regulatory sites in the *XCV3927* mRNA. It should be noted that the assessment of RNA stability by rifampicin treatment is hampered by the fact that our *Xcv* strains are rifampicin resistant.

The sX13 loops are reminiscent of regulatory RNAs in *S. aureus*, many of which contain ‘UCCC’-motifs in loops [66]. For example, RNAIII contains C-rich stem-loops, which interact with the RBS of target mRNAs [63], [65], [67]. RNAIII represses Rot synthesis through formation of kissing complexes between two stem-loops of each RNAIII and *rot* mRNA [64], [65]. Such multiple loop interactions are also employed by the *E. coli* sRNA OxyS to target *fhlA* [68]. In *Helicobacter pylori*, the sRNA HPnc5490 represses the synthesis of the chemotaxis regulator TlpB [69]. Interestingly, the central part of the HPnc5490-loop sequence is identical to the ‘UCCCCCU’-motif of loop 3 in sX13 [69].

### G-rich enhancer motifs confer sX13-dependency of target mRNAs

Similarly to RNAIII targets in *S. aureus* and the *tlpB* mRNA in *H. pylori* [65], [69], mRNAs repressed by sX13 are enriched for G-rich motifs in proximity of the TLS (Figure S4; Table S2). The complementarity between these motifs and the 4C-/5C-motif in the sX13 loops suggests sX13-mRNA interactions through antisense base pairing. Our data emphasize that sX13 acts posttranscriptionally on target genes that contain G-rich motifs, as shown for *XCV3927* and *pilH* (Figure S7). This idea is supported by the fact that mutation of the G-rich motifs, located in the mRNA of *XCV3927*, *pilH* and *hfq*, abolished sX13-dependency of protein synthesis (Figure 8; Figure S5; Figure S7). However, the presence of a G-rich motif does not necessarily confer regulation by sX13 (see *XCV0612*; Figure 7F; Figure 8D). Given that eight of 28 repressed and 4G-motif-containing mRNAs contain at least two 4G-motifs close to the TLSs (Figure S4), we assume that sX13 loops can interact with multiple 4G-motifs in certain mRNAs. As positively regulated mRNAs lack G-rich motifs, sX13 presumably acts indirectly on the corresponding genes (Figure S4; Table S2).

Direct sRNA-mRNA interactions are commonly validated by compensatory mutant studies [1]. However, in case of the *E. coli* sRNA RyhB, mutations were suggested to interfere with Hfq-binding and rendered compensatory mutants non-functional [70]. Here, mutation and deletion of *sX13* increased the synthesis of XCV3927::GFP and Hfq::GFP fusions, whereas mutation of corresponding 4G-motifs resulted in similar fluorescence values as non-mutated mRNA::*gfp* fusions in *Xcv* wild type. In addition, the reduced fluorescence of mutated target::GFP fusions was unaffected by compensatory sX13-mutant derivatives (Figure 8). This suggests that G-rich motifs in sX13-repressed mRNAs play a role besides mediation of sRNA interactions. While *Xanthomonas* spp., like other G+C-rich bacteria, lack a consensus RBS [16], [71], 5% of the chromosomal *Xcv* coding sequences (241 of 4,487) contain a G-rich motif 8–15 nucleotides upstream of their TLS (Figure S4). As anticipated, mutation of the 5G-motif at the RBS position of *pilH* abolished translation (Figure 8; Figure S5; Figure S7). By contrast, the 4G-motifs in *XCV3927* and *hfq* mRNAs, located 21 nucleotides upstream and nine nucleotides downstream of the AUG, respectively, confer sX13-dependency but were not essential for translation (Figure 8). Thus, G-rich motifs confer sX13-dependency and mRNA translation in a position-dependent manner. As mutation of the 4G-motif in *XCV3927* reduced protein synthesis, the motif appears to function as translational enhancer (Figure S7). We suggest that sequestration of a G-rich motif by sX13 as well as mutation of the motif precludes the binding of an unknown factor, which promotes mRNA translation. Such a factor could be RNA, protein or the ribosome.

The presumed sX13 mode of action is reminiscent of the *Salmonella* sRNA GcvB, which inhibits translation of mRNAs by targeting C/A-rich enhancer elements [72], [73]. By increasing the ribosome-binding affinity, C/A-rich motifs enhance mRNA translation, irrespective of their localization upstream or downstream of the TLS [72], [74].

### sRNAs encoded at the *polA* locus in other bacteria

Homologs of *Xcv* sRNAs are predominantly found in members of the *Xanthomonadaceae* family but not in other bacteria [15], [16]. The *sX13* gene is located adjacent to the DNA polymerase I-encoding *polA* gene, which resembles a locus encoding the Spot42 sRNA in *E. coli* and members of the αr7 sRNA family in α-proteobacteria [75], [76], [77], [78]. In contrast to sX13, Spot42 requires Hfq and regulates targets involved in carbon metabolism [48], [79], e. g., the discoordinated expression of genes within the *gal* galactose utilization operon [47], which is absent in *Xcv*. Although sX13 lacks sequence similarity to Spot42 and αr7 sRNAs, the latter also contain three stem-loops and apical C-rich motifs [80] suggesting that sRNAs in distantly related bacteria evolved divergently but retained structural conservation. Thus, it will be interesting to see whether the *polA* locus in other bacteria also encodes sRNAs, and whether sX13 and structurally related sRNAs act in a similar manner.

## Materials and Methods

### Bacterial strains and growth conditions

For bacterial strains, plasmids and oligonucleotides used in this study see Table S1. *E. coli* strains were grown at 37°C in lysogeny broth (LB), *Xcv* strains at 30°C in nutrient-yeast-glycerol (NYG) [81], XVM2 [40] or minimal medium A (MMA) [82], which was supplemented with casamino acids (0.3%) and sucrose (10 mM). Plasmids were introduced into *E. coli* by chemical transformation and into *Xcv* by tri-parental conjugation, using pRK2013 as helper plasmid [83]. Antibiotics were added to the final concentrations: ampicillin, 100 µg/ml; gentamycin, 15 µg/ml; kanamycin, 25 µg/ml; rifampicin, 100 µg/ml; spectinomycin, 100 µg/ml.

### Generation of constructs

To generate the sRNA-expression vector pBRS, a 28-bp fragment between the *lac* promoter and the *Eco*RI cloning site in pBBR1mod1 [84] was replaced by a truncated fragment, amplified by PCR from pBBR1mod1 using primers pBRS-EcoRI-fw and pBRS-NcoI-rev. For generation of constructs expressing sX13 (p*sX13*) and sX13Δ5′ (p*sX13Δ5′*; lacking 14 nt at the 5′-end), respective fragments were PCR-amplified from *Xcv* 85-10 using primers sX13-fw/-rev or sX13d5-fw/-rev. PCR products were digested with *Eco*RI/*Hin*dIII and cloned into pBRS. The *sX13*-mutant plasmids p*L1*, p*L2*, p*L3* and p*L2/3* were generated by PCR amplification of plasmid p*sX13* using primers L1-fw/-rev, L2-fw/-rev, L3-fw/-rev and L3-fw/L2/3-rev, respectively; plasmid p*L1/3* was generated with primers L3-fw/-rev and p*L1* as template; p*L1/2* was generated with primers L2-fw/L1/2-rev and p*L2* as template. For ectopic expression of *hfq* under control of its own promoter, a PCR product obtained from *Xcv* 85-10 using primers pMphfq-fw/-rev was cloned into the promoterless vector pBRM-P [84].

The GFP-based promoter-less reporter plasmid pFX-P permits *Bsa*I-mediated cloning of PCR amplicons (Golden Gate cloning) in a one-step restriction-ligation reaction [54] and was generated as follows: pDSK602 [85] was digested with *Pst*I/*Bam*HI to remove the *lac* promoter and multiple-cloning site. To allow blue-white selection, a dummy module containing 5′- and 3′-*Bsa*I recognition sites, p*lac* and *lacZ* was PCR-amplified from pBRM-P [84] using primers pFX-lz-fw/-rev. A fragment containing both the *gfp* coding sequence without translation start codon and the *rrnB* terminator was PCR-amplified from pXG-1 [53] using primers pFXgfp-fw/-rev. After blunt-end ligation of dummy- and *gfp*-module, the fragment was digested with *Mph1103*I/*Bgl*II and ligated into the *Pst*I/*Bam*HI sites of the pDSK602 backbone, resulting in pFX-P. For generation of the GFP-control plasmid pFX0, a promoterless fragment (138 bp) of the sRNA gene *sX6* [16] was PCR-amplified from *Xcv* 85-10 using primers pFX0-fw/-rev and cloned into pFX-P.

To generate mRNA::*gfp* expression constructs, fragments containing the promoter, 5′-UTR and 10 to 25 codons of the respective genes were PCR-amplified from *Xcv* 85-10 using corresponding pFX-fw/-rev primers (Table S1) and cloned into pFX-P. Plasmids pFX*3927*, pFX*hfq* and pFX*0612* were generated by cloning of nucleotide sequences −98 to +30, −160 to +75 and −116 to +33 relative to the translation start codon of *XCV3927*, *hfq* and *XCV0612*, respectively. pFX*pilH* was constructed by cloning a fragment spanning nucleotides −99 upstream of the *pilG* translation start codon to nucleotide +60 within the coding sequence of *pilH*.

The 4G-motif in *XCV3927::gfp* and *hfq::gfp* is located 21–24 bp upstream and 9–12 bp downstream of the ATG, respectively. *pilH::gfp* and *XCV0612::gfp* contain a 5G-motif at nucleotide positions 10–14 and 8–12 upstream of the ATG, respectively. Plasmids pFX*_MUT_* were constructed as follows: to mutate the ‘GGGG’ motif to ‘GCGC’, sequences upstream and downstream of the motif were PCR-amplified from *Xcv* 85-10 using primers pFX-fw/pFX-mut-L-rev and pFX-mut-R-fw/pFX-rev, respectively. Primers pFX-mut-L-rev and pFX-mut-R-fw contain the mutation flanked by a *Bsa*I-recognition site. pFX and corresponding pFX*_MUT_* derivatives only differ in the sequence of the G-rich motif at nucleotide positions relative to the translation start codons: −24/−22 in pFX*3927_MUT_*, +10/+12 in pFX*hfq_MUT_* and −12/−10 in pFX*pilH_MUT_*.

Plasmids pFXpl, which express p*lac*-driven mRNA::*gfp* fusions, were constructed by cloning respective fragments into pFX-P: p*lac* was PCR-amplified from pBRM-P [84] using primers plac-fw/rev; sequences −59 to +54 and −147 to +54 relative to the ATG of *hrpX* and *hrpG*, respectively, were PCR-amplified from *Xcv* 85-10 using primers pFXpl-hrpX-fw/-rev and pFXpl-hrpG-fw/-rev; fragments of *XCV3927* and *pilH* were PCR-amplified from respective pFX and pFX*_MUT_* plasmids using primers pFXpl3927-fw/pFXpl3927mut-fw/pFX3927-rev and pFXplpilH-fw/pFXpilH-rev.

### Generation of *Xcv* mutant strains

To generate a chromosomal *sX13* deletion mutant, flanking sequences of ∼650 bp up- and downstream of *sX13* [16] were amplified by PCR from *Xcv* 85-10 using primers d13L-fw/-rev and d13R-fw/-rev. PCR products were digested with *Bam*HI/*Hin*dIII and *Hin*dIII/*Xba*I, respectively, and ligated into the suicide vector pOK1 [86]. *Xcv*Δ*sX13*+*sX13*_ch_, which carries an *sX13* copy at the Δ*sX13* locus, was created as follows: two PCR fragments amplified from *Xcv* 85-10 using primers int13L-fw/rev and int13R-fw/rev were digested with *Psp*1406I, ligated and cloned into the *Bam*HI/*Xba*I site of pOK1. Conjugation of pOKΔ*sX13* into *Xcv* 85-10 and pOKint13 into *Xcv*Δ*sX13* and selection of the correct double crossing-over events were performed as described [86]. *Xcv*Δ*sX13*+*sX13*_ch_ was identified by PCR amplification of the *sX13* locus and *Psp*1406I restriction.

To introduce a frameshift mutation into chromosomal *hfq*, PCR products obtained from *Xcv* 85-10 using primers hfqL-fw/-rev and hfqR-fw/-rev were digested with *Bam*HI/*Bsa*I and *Bsa*I/*Xba*I, respectively, and cloned into pOK1. Conjugation of pOK-fs*hfq* into *Xcv* and selection of double crossing-over events were performed as described [86]. The resulting *hfq* mutant strain carries a 4 bp deletion in an *Mnl*I site (nucleotides 33–36 in *hfq*) and was identified by PCR using primers seqhfq-fw/-rev followed by digestion with *Mnl*I.

### Plant material and plant inoculations

Pepper (*Capsicum annuum*) plants of the near-isogenic cultivars ECW and ECW-10R [87] were grown at 25°C with 60–70% relative humidity and 16 hours light. For infection assays, *Xcv* bacteria were resuspended in 10 mM MgCl_2_ and inoculated with a needleless syringe into the intercellular spaces of leaves using concentrations of 1–4×10^8^ colony-forming units (CFU) per ml for scoring plant reactions and 10^4^ CFU/ml for *in planta* growth curves. For better visualization of the HR, leaves were bleached in 70% ethanol. *In planta* growth curves were performed as described [33]. All experiments were repeated at least two times.

### Protein detection and measurement of GFP fluorescence in *Xcv*

*Xcv* cells grown overnight in NYG medium were washed, incoculated at OD_600_ = 0.2 into XVM2 medium and incubated for 3.5 hours at 30°C. Total cell extracts were analyzed by sodium dodecyl sulfate-polyacrylamide gel electrophoresis and immunoblotting using specific polyclonal antibodies directed against HrpF [88], HrcN [89], HrcJ [89] and GroEL (Stressgen). A horseradish peroxidase-labeled anti-rabbit antibody (Amersham Pharmacia Biotech) was used as secondary antibody. Antibody reactions were visualized by enhanced chemiluminescence (Amersham Pharmacia Biotech).

To determine GFP fluorescence, bacteria were adjusted to OD_600_ = 1.0 in 10 mM MgCl_2_. Fluorescence was measured at 485-nm excitation and 535-nm emission using a microplate reader (SpectraFluor Plus; Tecan Trading AG).

### *In vitro* transcription and structure probing

*sX13* [16] was PCR-amplified from *Xcv* 85-10 using primers sX13T7-fw, containing the T7-promoter, and sX13T7-rev and cloned into pUC57 (Thermo Fisher Scientific), resulting in pUC-13T7. Template DNA for *in vitro* transcription was amplified from pUC-13T7 using primers sX13-ITC-fw/-rev. *sX13* transcription and DNase treatment were performed according to manufacturer's instructions (MEGAscript®Kit; Invitrogen). RNA labeling using [γ-^32^P]-ATP, treatment with RNase T1 (1 Pharmacia unit; Ambion) or RNase V1 (0.01 to 0.0002 Pharmacia units; Ambion) and generation of nucleotide ladders were performed as described [90]. Samples were analyzed on 12% polyacrylamide gels containing 7 M urea. Signals were visualized with a phosphoimager (FLA-3000 Series; Fuji).

### RNA preparation, Northern blot and qRT-PCR analysis

Bacteria were grown overnight in NYG and inoculated at OD_600_ = 0.2 into NYG or XVM2 medium. XVM2 cultures were incubated for 3.5 hours at 30°C. NYG-grown cells were harvested at exponential growth phase (OD_600_ = 0.5–0.7) or used to inoculate the following media at OD_600_ = 0.5: NYG containing 0.3 M NaCl, 0.2 M H_2_O_2_ or NYG lacking a nitrogen source, MMA or MMA lacking a carbon source followed by incubation for 3 hours.

RNA isolation and Northern blot hybridization was performed as described [16], [91]. Oligonucleotide probes for detection of sX13 and 5S rRNA are described in [16].

For qRT-PCR analyses, cDNA was synthesized using RevertAid H Minus First Strand cDNA-Synthesis Kit according to manufacturer's instructions (Fermentas). qRT-PCR was performed using 2 ng cDNA and ABsolute BlueSYBR Green Fluorescein (Thermo Scientific) and analyzed on MyiQ2 and CFX Connect systems (Bio-Rad). The efficiency and specificity of PCR amplifications was determined by standard curves derived from a dilution series of template cDNA and melting curve analysis, respectively. Mean transcript levels were calculated based on values obtained from technical duplicates of at least three independent biological replicates and the levels of 16S rRNA (reference gene) as described (ABI user bulletin 2; Applied Biosystems).

### Microarray analysis

For isolation of total RNA, NYG-grown cells were harvested at exponential growth phase (OD_600_ = 0.5–0.7) or used to inoculate MMA at OD_600_ = 0.5 followed by incubation for 3 hours. Fluorescently labeled cDNA was prepared as described [92]. Starting from 10–15 µg total RNA, aminoallyl-modified first strand cDNA was synthesized by reverse transcription using random hexamer primers, Bioscript reverse transcriptase (Bioline) and 0.5 mM dNTP, dTTP∶aminoallyl-dUTP (1∶4). After hydrolysis and clean up using Nucleotide removal kit (Qiagen), Cy3- and Cy5-N-Hydroxysuccinimidyl ester dyes (GE Healthcare) were coupled to the aminoallyl-labeled first strand cDNA. Uncoupled dye was removed using the Nucleotide removal kit (Qiagen). For RNA from NYG- and MMA-grown bacteria, four and three microarray hybridizations were performed, respectively, using labeled cDNA obtained from independent bacterial cultures.

The genome-wide microarray for *Xcv* strain 85-10 (Xcv5KOLI) carried 50–70 nt unique oligonucleotides representing CDSs, with each oligonucleotide spotted in three technical replicates per microarray [93]. Preprocessing of microarrays was performed as described [94]. Hybridization was performed in EasyHyb hybridization solution (Roche) supplemented with sonicated salmon sperm DNA at 50 µg/ml in a final volume of 130 µl for 90 min at 45°C using the HS400 Pro hybridization station (Tecan Trading AG). Labeled cDNA samples were denatured for 5 min at 65°C prior hybridization. After hybridization microarrays were washed as described [94].

Mean signal and mean local background intensities were obtained for each spot on the microarray images using ImaGene 8.0 software for spot detection, image segmentation and signal quantification (Biodiscovery Inc.). Spots were flagged as empty if *R*≤0.5 in both channels, where *R* = (signal mean−background mean)/background standard deviation. Remaining spots were analyzed further. The log_2_ value of the ratio of intensities was calculated for each spot using the formula M_i_ = log_2_(*R*_i_/*G*_i_). *R*_i_ = *I*_ch1(i)_-*Bg*_ch1(i)_ and *G*_i_ = *I*_ch2(i)_-*Bg*_ch2(i)_, where *I*_ch1(i)_ or *I*_ch2(i)_ is the intensity of a spot in channel 1 or channel 2, and *Bg*_ch1(i)_ or *Bg*_ch2(i)_ is the background intensity of a spot in channel 1 or channel 2. The mean intensity was calculated for each spot, *A*_i_ = log_2_(*R*_i_ *G*_i_)^0.5^ [95]. For data normalization (Median), significance test (Holm) and *t*-statistics analysis, the EMMA 2.8.2 open source platform was used [49]. Genes were accounted as differentially expressed if *P adjusted* ≤0.05, *A*≥8, and if the ratio showed at least a 1.5-fold difference between the two experimental conditions.

### Biocomputational analyses

Homology searches were performed using BLASTn and the NCBI genome database (http://blast.ncbi.nlm.nih.gov; http://www.ncbi.nlm.nih.gov/genome; date: 11/22/2012).

The secondary structure of sX13 [16] was predicted using Mfold version 3.5 (http://mfold.rna.albany.edu/?q=mfold/RNA-Folding-Form) and default folding parameters [55]. To identify putative regulatory motifs in the 5′-regions of sX13-regulated mRNAs, a discriminative motif search was performed using DREME version 4.9.0 (http://meme.nbcr.net/meme/cgi-bin/dreme.cgi) [56]. Sequences of regulated genes comprising nucleotide positions −100 to +100 relative to translation start codons or in case of known TSSs [16] (see Table S2), sequences comprising the 5′-UTR to position +100 downstream of translation start codons, were extracted from the genome of *Xcv* strain 85-10 (NC_007508 and NC_007507) [32]. DREME motif search was performed with negatively regulated genes as input and positively regulated genes as comparative sequences and an *E*-value of ≤5.

### Accession numbers

YP_363045.1; YP_363046.1; YP_362142.1; YP_362163.1; YP_362160.1; YP_361663.1; YP_363499.1; YP_365931.1; YP_363887.1; YP_365930.1; YP_365658.1; YP_364552.1; YP_363772.1; YP_363917.1; YP_364964.1; YP_364963.1; YP_364545.1; YP_365303.1; YP_365304.1; YP_362343.1; YP_362302.1; YP_362409.1; YP_364550.1; YP_364798.1; YP_364827.1; YP_365231.1; YP_363688.1; YP_363753.1; YP_361904.1; YP_363264.1; YP_362055.1; YP_363957.1

## Supporting Information

Figure S1 sX13 abundance is not affected by expression of HrpG*. *Xcv* 85-10 (wt), Δ*sX13* and Δ*sX13*+*sX13*_ch_ and strains additionally expressing HrpG* were incubated for 3.5 hours in *hrp*-gene inducing medium XVM2 (see Figure 2B). Total RNA was analyzed by Northern blot using an sX13-specific probe. 5S rRNA was probed as loading control. The experiment was performed twice with similar results.
(EPS)

Figure S2 **Structure probing of sX13.** *In vitro* transcribed sX13 was 5′-labeled and treated with RNase T1 (T1) or alkaline hydroxyl (OH^−^) buffer for generation of nucleotide ladders and RNase V1 (V1) for mapping of base-paired regions. Lane ‘C’ contains untreated sX13; triangle indicates decreasing concentrations of RNase V1; ‘#G’ indicates positions of G residues; the deduced secondary structure is indicated on the right hand side (see Figure 6A).
(EPS)

Figure S3 **Expression of sX13 derivatives.** Total RNA of NYG-grown *Xcv* strains 85-10 (wt) and Δ*sX13* carrying pBRS (pB), p*sX13* or expressing mutated sX13-derivatives (see Figure 6) was analyzed by Northern blot using an sX13-specific probe. 5S rRNA was probed as loading control. The experiment was performed twice with similar results.
(EPS)

Figure S4 **Distribution of 4G-motifs among sX13-regulated genes and chromosomally encoded** ***Xcv*** **genes.** (A) Percentage of sX13-regulated genes identified by microarray analyses (see Table S2) and chromosomal CDSs in *Xcv* containing one or more 4G-motifs in region −100 to +100 relative to the TLS or in case of known TSSs, in the sequence comprising the 5′-UTR to position +100. The number of genes analyzed (n) is given below. (B) Distribution of 4G-motifs found in region −100 to +100 bp relative to the TLSs of 1,378 chromosomal CDSs [see (A)].
(EPS)

Figure S5 **sX13-dependency of mRNA target::GFP synthesis in MMA-grown** ***Xcv*** **strains.** GFP fluorescence of MMA-grown *Xcv* strains 85-10 (wt) and Δ*sX13* carrying pB or p*sX13* and carrying GFP-reporter plasmids pFX*3927*, pFX*3927_MUT_*, pFX*pilH* or pFX*pilH_MUT_*. pFX*3927_MUT_* and pFX*pilH_MUT_* contain a mutated 4G- and 5G-motif, respectively. *Xcv* autofluorescence was determined using pFX0. GFP fluorescence of the wt was set to 1. Data points and error bars represent mean values and standard deviations obtained from three independent experiments. Statistically significant differences compared to the wt are indicated by an asterisk (*t*-test; *P*<0.03). For comparison, see Figure 8A and C.
(EPS)

Figure S6 **mRNA amount of** ***XCV3927::gfp*** **is sX13-independent.** The *XCV3927::gfp* mRNA amount in NYG-grown *Xcv* strains 85-10 (wt) and Δ*sX13* carrying pB, p*sX13* or mutated sX13-derivatives and containing pFX*3927* or pFX*3927_MUT_* was analyzed by qRT-PCR using *gfp*-specific oligonucleotides. The RNA level in the wt was set to 1. Data points and error bars represent mean values and standard deviations obtained with three independent biological samples. For comparison, see Figure 7B and Figure 8A.
(EPS)

Figure S7 **sX13 posttranscriptionally affects XCV3927::GFP and PilH::GFP synthesis.** GFP fluorescence of NYG-grown *Xcv* strains 85-10 (wt), Δ*sX13* and Δ*sX13* containing chromosomally re-integrated *sX13* (Δ*sX13*+*sX13*_ch_); strains express *XCV3927::gfp* (pFXpl-3927) or *pilH::gfp* (pFXpl-*pilH*) under control of p*lac*. pFX*_MUT_* derivatives contain a mutated 4G-motif. *Xcv* autofluorescence was determined using pFX0 and is indicated by dashed line. GFP fluorescence of the wt carrying pFXpl-*3927* or pFXpl-*pilH* was set to 1. Data points and error bars represent mean values and standard deviations obtained from three independent experiments. Asterisks indicate statistically significant differences (*t*-test; *P*<0.03).
(EPS)

Figure S8 **Translation of HrpG::GFP and HrpX::GFP is sX13-independent.** GFP fluorescence of NYG-grown *Xcv* strains 85-10 (wt) and Δ*sX13* expressing *hrpG::gfp* (pFXpl-*hrpG*) or *hrpX::gfp* (pFXpl-*hrpX*) under control of p*lac*. *Xcv* autofluorescence was determined using pFX0 and is indicated by dashed line. GFP fluorescence of the wt was set to 1. Data points and error bars represent mean values and standard deviations obtained from three independent experiments. Differences were not statistically significant (*t*-test; *P*<0.03).
(EPS)

Table S1 **Bacterial strains, plasmids and oligonucleotides used in this study.**
(PDF)

Table S2 **sX13-regulated genes identified by microarray and qRT-PCR analysis.**
(PDF)
