# Supplementary material for: Small RNA sX13: A Multifaceted Regulator of Virulence in the Plant Pathogen Xanthomonas
Source: PLoS Pathog. 2013 Sep 12;9(9):e1003626. doi: 10.1371/journal.ppat.1003626 (PMC3771888; doi:10.1371/journal.ppat.1003626)
Supplement: Table S1 — Bacterial strains, plasmids and oligonucleotides used in this study. (PDF) [file ppat.1003626.s009.pdf]

Table S1. Bacterial strains, plasmids and oligonucleotides used in this study.

**Strain or plasmid**	**Relevant characteristics**[a]	**Reference or source**
Xanthomonas campestris* pv. *vesicatoria		
85-10	Pepper-race 2; wild type; Rif^{R}	[1]
Δ*sX13*	85-10 derivative deleted in *sX13*; Rif^{R}	This study
Δ*sX13*+*sX13*$_{ch}$	Δ*sX13* derivative containing re-integrated *sX13* at Δ*sX13* locus; Rif^{R}	This study
hfq^{-}	*hfq* frameshift mutant of strain 85-10; Rif^{R}	This study
Δ*sX13hfq*$^{-}$	85-10 derivative deleted in *sX13* and containing frameshift mutation in *hfq*; Rif^{R}	This study
Escherichia coli		
DH5α λpir	F^{-} *recA hsdR17(r$_{k}^{-}$,m$_{k}^{+}$) ϕ80dlacZ ΔM15 [λpir]*	[2]
TOP10	F^{-} *mcr*A Δ(*mrr*-*hsd*RMS-*mcr*BC) ϕ80*lacZ*ΔM15 Δ*lac*X74 *rec*A1 *ara*D139 Δ(*ara-leu*) 7697 *gal*U *gal*K *rps*L (Str^{R}) *end*A1 *nup*G	Invitrogen
**Plasmids**		
pRK2013	ColE1 replicon, TraRK^{+} Mob^{+}; Km^{R}	[3]
pFG72-1	Derivative of pUFR043 containing *hrpG**; Km^{R}	[4]
pOK1	Suicide vector; *sacB sacQ mobRK2* oriR6K; Sm^{R}	[5]
pOKΔ*sX13*	pOK1 derivative containing flanking regions of *sX13*; Sm^{R}	This study
pOKint13	pOK1 derivative containing *sX13* locus; Sm^{R}	This study
pOK-fs*hfq*	pOK1 derivative for frameshift mutation of *hfq*; Sm^{R}	This study
pBRM-P	pBBR1MCS-5 derivative without promoter; Gm^{R}	[6]
p*hfq*	pBRM-P derivative containing *hfq*; Gm^{R}	This study
pBBR1mod1	pBBR1MCS-5 derivative without polylinker; Gm^{R}	[6]
pBRS	pBBR1mod1 derivative for sRNA expression; Gm^{R}	This study
p*sX13*	pBRS derivative expressing *sX13*; Gm^{R}	This study
p*sX13Δ5'*	pBRS derivative expressing *sX13Δ5* (lacks 14 nt at 5' end); Gm^{R}	This study
p*L1*	p*sX13* derivative containing mutations in *sX13* loop 1; Gm^{R}	This study
p*L2*	p*sX13* derivative containing mutations in *sX13* loop 2; Gm^{R}	This study
p*L3*	p*sX13* derivative containing mutations in *sX13* loop 3; Gm^{R}	This study
pL1/2	p*L2* derivative containing mutations in *sX13* loops 1 and 2; Gm^{R}	This study
p*L1/3*	p*L1* derivative containing mutations in *sX13* loops 1 and 3; Gm^{R}	This study
p*L2/3*	p*sX13* derivative containing mutations in *sX13* loops 2 and 3; Gm^{R}	This study
pDSK602	Broad-host-range vector; contains triple *lacUV5* promoter; Sm^{R}	[7]
pXG-1	GFP expression plasmid; Cm^{R}	[8]
pFX-P	Golden Gate-compatible pDSK602 derivative without promoter for generation of translational *mRNA::gfp* fusions; Sm^{R}	This study
pFX0	Promoterless pFX-P derivative; control plasmid for GFP reporter fusions (measurement of *Xcv* autofluorescence); Sm^{R}	This study
pFX*3927*	pFX-P derivative for expression of XCV3927::GFP; Sm^{R}	This study
pFX*hfq*	pFX-P derivative for expression of Hfq::GFP; Sm^{R}	This study
pFX*pilH*	pFX-P derivative for expression of PilH::GFP; Sm^{R}	This study
pFX*0612*	pFX-P derivative for expression of XCV0612::GFP; Sm^{R}	This study
pFX*3927*$_{MUT}$	pFX-P derivative containing mutation in 4G-motif within 5'-UTR of *XCV3927*; Sm^{R}	This study
pFX*hfq*$_{MUT}$	pFX-P derivative containing mutation in 4G-motif within CDS of *hfq*; Sm^{R}	This study
pFX*pilH*$_{MUT}$	pFX-P derivative containing mutation in 5G-motif within 5'-UTR of *pilH*; Sm^{R}	This study
pFXpl-*3927*	pFX-P derivative for p*lac*-driven expression of XCV3927::GFP; Sm^{R}	This study
pFXpl-*pilH*	pFX-P derivative for p*lac*-driven expression of PilH::GFP; Sm^{R}	This study
pFXpl-*3927*$_{MUT}$	pFX-P derivative containing p*lac* and mutation in 4G-motif within 5'-UTR of *XCV3927*; Sm^{R}	This study
pFXpl-*pilH*$_{MUT}$	pFX-P derivative containing p*lac* and mutation in 5G-motif within 5'-UTR of *pilH*; Sm^{R}	This study
pFXpl-*hrpG*	pFX-P derivative for p*lac*-driven expression of HrpG::GFP; Sm^{R}	This study
pFXpl-*hrpX*	pFX-P derivative for p*lac*-driven expression of HrpX::GFP; Sm^{R}	This study
pUC-13T7	pUC57 derivative containing T7 promoter upstream of *sX13*; Ap^{R}; Sm^{R}	This study
**Oligonucleotide**	**Sequence**[b]	**Purpose**
pBRS-EcoRI-fw	AACCTTAAGATTCCACACAACATACGAGC	Generation of pBRS
pBRS-NcoI-rev	CGTCCATGGGCAAATATTATA	Generation of pBRS
sX13-fw	TCAGAATTCGCGCAACGCCTGTCGGTAGA	Generation of p*sX13*
sX13-rev	GCTAAGCTTGCGCATAGTGGAAGGACACAAAT	Generation of p*sX13*
sX13d5-fw	TGGGAATTCGATCTCTCCCATCCCCTGG	Generation of p*sX13Δ5*

Oligonucleotide	Sequence[b]	Purpose
sX13d5-rev	TGGAAGCTTATAAAAAGCCCCGCAGACCAG	Generation of p*sX13*Δ*5*
L1-fw	CGGAAACTCCTCCCCAAGTTT	Generation of p*L1*
L1-rev	CTCCGAGATCTGCTCCAGCGCATGGGAG	Generation of p*L1*
L2-fw	AGCGGAAACTCCTGCGCAAGTTTCCGTTCC	Generation of p*L2*
L2-rev	CCGAGATCTGCTCCAGGGGATG	Generation of p*L2*
L3-fw	CCCCGCCGACCTGCGCCTGGTCTGC	Generation of p*L3*
L3-rev	CCAGGGAACGGAAACTTGGGGA	Generation of p*L3*
L1/2-rev	CCGAGATCTGCTCCAGCGCATGGGAGAGATC	Generation of p*L1/2*
L2/3-rev	CCAGGGAACGGAAACTTGCGCAGGAGTTTCC	Generation of p*L2/3*
plac-fw	TTTGGTCTCTATTCTGAGCGCAACGCAATTAATG	Generation of pFXpl
plac-rev	TTTGGTCTCTCCACCCACACAACATACGAGCCGG	Generation of pFXpl
pFX-lz-fw	GACATGCATGAATTCAGAGACCGCAGCTG	Generation of pFX-P
pFX-lz-rev	Phosphate-AGAGACCTTACAATTTCCATTCGC	Generation of pFX-P
pFXgfp-fw	Phosphate-GCTAGCAAAGGAGAAGAACTTTTCACTG	Generation of pFX-P
pFXgfp-rev	GACAGATCT AGCAAAACCCGTACCCTAGGTC	Generation of pFX-P
pFX0-fw	TTTGGTCTCTATTCCGCGAGGAAGAGGAAGAAGAA	Generation of pFX0
pFX0-rev	TTTGGTCTCTTAGCCATACAGCTACCCCAAAAGCGAAC	Generation of pFX0
pFX3927-fw	TTTGGTCTCTATTCCGGCAAGACGCTGTCATTCTAG	Generation of pFX*3927*
pFX3927-rev	TTTGGTCTCTTAGCAGCGACGACCGTACGAAGTC	Generation of pFX*3927*
pFXhfq-fw	TTTGGTCTCTATTCAGCGTGACCGCCATCAATTG	Generation of pFX*hfq*
pFXhfq-rev	TTTGGTCTCTTAGCATACACCGACACGGGCACC	Generation of pFX*hfq*
pFXpilH-fw	TTTGGTCTCTATTCACCCAGACGTGGTCGGAAC	Generation of pFX*pilH*
pFXpilH-rev	TTTGGTCTCTTAGCCCATTGACTGAAGACTGCCCTG	Generation of pFX*pilH*
pFX0612-fw	TTTGGTCTCTATTCATCGCGTGGTTTGTGATAAGTG	Generation of pFX*0612*
pFX0612-rev	TTTGGTCTCTTAGCCACCAGCGCTCTTAGTTGTTCTG	Generation of pFX*0612*
pFX3927mut-L-rev	TTTGGTCTCTGCGCAACAGGTCTGCGCACTATAGTCTAG	Generation of pFX*3927*$_{MUT}$
pFX3927mut-R-rev	TTTGGTCTCTGCGCAATCAGGCAAGAAGGCACCTATG	Generation of pFX*3927*$_{MUT}$
pFXhfqmut-L-rev	TTTGGTCTCTGCGCTTAGCCATCGAAAAATCCTCTTCA	Generation of pFX*hfq*$_{MUT}$
pFXhfqmut-R-rev	TTTGGTCTCTGCGCCAATCTTTACAGGACCCATTCCTC	Generation of pFX*hfq*$_{MUT}$
pFXpilHmut-L-rev	TTTGGTCTCTGCGCCTGGTCAGGCGTGGACGTAC	Generation of pFX*pilH*$_{MUT}$
pFXpilHmut-R-rev	TTTGGTCTCTGCGCAAAGGCAACATGGCTCGAATTATAT	Generation of pFX*pilH*$_{MUT}$
pFXpl3927-fw	TTTGGTCTCTGTGGACCTGTTGGGGAATCAGGCA	Generation of pFXpl-*3927*
pFXpl3927mut-fw	TTTGGTCTCTGTGGACCTGTTGCGCAATCAGGCA	Generation of pFXpl-*3927*$_{MUT}$
pFXplpilH-fw	TTTGGTCTCTGTGGGTTTCGTAGCGACGTCGGAAG	Generation of pFXpl-*pilH*
pFXpl-hrpG-fw	TTTGGTCTCTGTGGGTCCAGCTCCACTGGACTCTC	Generation of pFXpl-*hrpG*
pFXpl-hrpG-rev	TTTGGTCTCTTAGCGTCCTGCGTCAACAGGAACAC	Generation of pFXpl-*hrpG*
pFXpl-hrpX-fw	TTTGGTCTCTGTGGCGCCAGCGAGTTCGGCGC	Generation of pFXpl-*hrpX*
pFXpl-hrpX-rev	TTTGGTCTCTTAGCACGTTCTGCGTATGACAACGCA	Generation of pFXpl-*hrpX*
d13L-fw	CAGGATCCGCTGGGAGTACGGCTTCACG	Deletion of *sX13*
d13L-rev	AACAAGCTTATTTGTGTCCTTCCACTATGCGCA	Deletion of *sX13*
d13R-fw	AACAAGCTTATTGATGGATCGTGAAGATAACTG	Deletion of *sX13*
d13R-rev	GCTCTAGAAACTTCGGCCTGATGTACG	Deletion of *sX13*
int13L-fw	CAGGATCCCCGAGAGCATCCTGATGAGTTT	Δ*sX13* complementation
int13L-rev	TGCAACGTTAACAGCGATGCTGCAGGTG	Δ*sX13* complementation
int13R-fw	GTTAACGTTGCAGCGCTTGCGCATAGTG	Δ*sX13* complementation
int13R-rev	GCTCTAGAAGCTGATCGCCTGCGACTATT	Δ*sX13* complementation
hfqL-fw	TCAGGATCCAAATTGCCGATTCTGGCCGG	Mutation of *hfq*
hfqL-rev	TTTGGTCTCTCATTATGGGTCCTGTAAAGATTGCCC	Mutation of *hfq*
hfqR-fw	TTTGGTCTCTAATGCGCTGCGGCGCGAGC	Mutation of *hfq*
hfqR-rev	GCATCTAGAGCGTGGCGAACAATTGATCT	Mutation of *hfq*
seqhfq-fw	GAGCGTGACCGCCATCAATTG	Screening *hfq* mutation
seqhfq-rev	GAACTCCTCCATCACATCGTCTTCG	Screening *hfq* mutation
pMphfq-fw	TTTGGTCTCTATTCAGCGTGACCGCCATCAATTG	Generation of p*hfq*
pMphfq-rev	CAGGGTCTCTCACCTTACTGCTCGACGTCGTCATCTTCCG	Generation of p*hfq*
sX13T7-fw	GAAATTAATACGACTCACTATAGGGCGCAACGCCTGTC	*in vitro* transcription
sX13T7-rev	TTATAAAAAGCCCCGCAGACCAGG	*in vitro* transcription
sX13-ITC-fw	TAATACGACTCACT	*in vitro* transcription
sX13-ITC-rev	ATAAAAAGCCCCGCA	*in vitro* transcription

Oligonucleotide	Sequence[b]	Purpose
q-16S-fw	TACGCTAATACCGCATACGAC	qRT-PCR
q-16S-rev	TGGCACGAAGTTAGCCGGTG	qRT-PCR
q-sX13-fw	CGCAACGCCTGTCGGTAGATCTC	qRT-PCR
q-sX13-rev	GGCCAGGGAACGGAAACTTG	qRT-PCR
q-gfp-fw	CCATGGCCAACACTTGTCACTA	qRT-PCR
q-gfp-rev	CAATGTTGTGGCGAATTTTGAA	qRT-PCR
q-algR-fw	ATCCGCAGGTCGAGGTGAT	qRT-PCR
q-algR-rev	ACCGGTTTCATCAGGTAATCCAG	qRT-PCR
q-asnB2-fw	GCCTACAACGGCGAGGTCTAT	qRT-PCR
q-asnB2-rev	ATCAGCTTGAAGGTGTGCTCGT	qRT-PCR
q-avrBs1-fw	AGGTCGCCACTCAGCAAGATAG	qRT-PCR
q-avrBs1-rev	TAGTCACCTCTTGGGGGTTTGA	qRT-PCR
q-cheY-fw	AGCGCACGTATCTTGGTGGT	qRT-PCR
q-cheY-rev	GCCTTGCCTTCGGATTTCTT	qRT-PCR
q-fliC-fw	GCACAGGTAATCAACACCAACG	qRT-PCR
q-fliC-rev	AGTTAGTTGCTTTCGCCGACTG	qRT-PCR
q-hfq-fw	ATGGCTAAGGGGCAATCTTTACAGG	qRT-PCR
q-hfq-rev	CGTCGTCATCTTCCGCCTGAT	qRT-PCR
q-hrcJ-fw	ATCAGGTGTCCTATTCGCTGGA	qRT-PCR
q-hrcJ-rev	TTTCGTAGGTGAGTCCCTCCAC	qRT-PCR
q-hrpF-fw	CAAGTCGGAGCTTCAGATCGTT	qRT-PCR
q-hrpF-rev	CTCCAGTTCGGATTGATTGAGC	qRT-PCR
q-hrpG-fw	TCTCGACGTTTTCCGATGAA	qRT-PCR
q-hrpG-rev	CATCGCGGATCAGCTTGTAC	qRT-PCR
q-hrpX-fw	GATGAGGTCAGCTTGTTCGGTG	qRT-PCR
q-hrpX-rev	GTCTGTAAGGCCAACGTGCTCTG	qRT-PCR
q-pilE-fw	TAGCGGAGCGATTTCACACA	qRT-PCR
q-pilE-rev	TCTGACTGGAGCCCCTTTGA	qRT-PCR
q-pilG-fw	AACTCGCAGGACTGAAGGTGAT	qRT-PCR
q-pilG-rev	GAACGGCTTGGTCAGATATTGC	qRT-PCR
q-pilH-fw	ACCGACAGGGCAGTCTTCAGT	qRT-PCR
q-pilH-rev	AACGCACCAATTGTCTGATGC	qRT-PCR
q-pilN-fw	TCTGACCACCGAGATCGACA	qRT-PCR
q-pilN-rev	GACCTTCCAGCGTGAGGATG	qRT-PCR
q-pilU-fw	AGAAGGCCTCGGACCTGTTC	qRT-PCR
q-pilU-rev	ACCATGCCCACCTGATTACG	qRT-PCR
q-xopJ-fw	CACGTCTTCTTACTCGGCCACT	qRT-PCR
q-xopJ-rev	CACGCTGGGAAACTACTGAGGT	qRT-PCR
q-xopS-fw	GCGATCATTCTGGAAGACCAGT	qRT-PCR
q-xopS-rev	TTGGCTTCAATCCTCGTCAGTT	qRT-PCR
q-XCV0173-fw	CCAAGCCCAAGGAGTTCTATTTCGAC	qRT-PCR
q-XCV0173-rev	CCAGGTGATGGCCCGGTACTG	qRT-PCR
q-XCV0612-fw	ATGAGGCGCATTTCTATGCTGT	qRT-PCR
q-XCV0612-rev	AATCAGTACGTCGAGGCGAATC	qRT-PCR
q-XCV2186-fw	AAGACTTTGTGCGCATTTCCAC	qRT-PCR
q-XCV2186-rev	AATCGGGTACTTCCTGCTTGGT	qRT-PCR
q-XCV2819-fw	TTGGATGCGTCAACGATCAG	qRT-PCR
q-XCV2819-rev	CGTCCATCGTCAACGTCGTA	qRT-PCR
q-XCV2821-fw	AGGACCGAGGCCATACGGAATG	qRT-PCR
q-XCV2821-rev	CGGCTGAAGAGTGACTTTCTGGTCTG	qRT-PCR
q-XCV3096-fw	TCCCTGCTACTGGCGTTGTT	qRT-PCR
q-XCV3096-rev	GATTCCGTTCGACCGTCTTG	qRT-PCR
q-XCV3572-fw	GTTGGTGCTGGTGAACGGTA	qRT-PCR
q-XCV3572-rev	ATATTCGGCAGACAGCGTCA	qRT-PCR
q-XCV3573-fw	GCAGCGACATTCACCTTGTG	qRT-PCR
q-XCV3573-rev	AGGTACAGCCGTGCACGCTG	qRT-PCR
q-XCV3927-fw	CCGCAAGGGCGATACCTTGTG	qRT-PCR
q-XCV3927-rev	CCATGTCCAGATCTTCGTTGTGCTTG	qRT-PCR

[a], Ap, ampicillin; Gm, gentamycin; Km, kanamycin; Rif, rifampicin; Sm, spectinomycin; [R], resistance. [b], Recognition sites of restriction enzymes are underlined.

References

1. Canteros BI (1990) Ph.D. thesis. University of Florida, Gainesville, FL.
2. Menard R, Sansonetti PJ, Parsot C (1993) Nonpolar mutagenesis of the *ipa* genes defines IpaB, IpaC, and IpaD as effectors of *Shigella flexneri* entry into epithelial cells. J Bacteriol 175: 5899-5906.
3. Figurski DH, Helinski DR (1979) Replication of an origin-containing derivative of plasmid RK2 dependent on a plasmid function provided *in trans*. Proc Natl Acad Sci U S A 76: 1648-1652.
4. Wengelnik K, Rossier O, Bonas U (1999) Mutations in the regulatory gene *hrpG* of *Xanthomonas campestris* pv. vesicatoria result in constitutive expression of all *hrp* genes. J Bacteriol 181: 6828-6831.
5. Huguet E, Hahn K, Wengelnik K, Bonas U (1998) *hpaA* mutants of *Xanthomonas campestris* pv. *vesicatoria* are affected in pathogenicity but retain the ability to induce host-specific hypersensitive reaction. Mol Microbiol 29: 1379-1390.
6. Szczesny R, Jordan M, Schramm C, Schulz S, Cogez V, et al. (2010) Functional characterization of the Xcs and Xps type II secretion systems from the plant pathogenic bacterium *Xanthomonas campestris* pv. *vesicatoria*. New Phytol 187: 983-1002.
7. Murillo J, Shen H, Gerhold D, Sharma A, Cooksey DA, et al. (1994) Characterization of pPT23B, the plasmid involved in syringolide production by *Pseudomonas syringae* pv. *tomato* PT23. Plasmid 31: 275-287.
8. Urban JH, Vogel J (2007) Translational control and target recognition by *Escherichia coli* small RNAs *in vivo*. Nucleic Acids Res 35: 1018-1037.
