# Supplementary material for: Small RNA sX13: A Multifaceted Regulator of Virulence in the Plant Pathogen Xanthomonas
Source: PLoS Pathog. 2013 Sep 12;9(9):e1003626. doi: 10.1371/journal.ppat.1003626 (PMC3771888; doi:10.1371/journal.ppat.1003626)
Supplement: Table S2 — sX13-regulated genes identified by microarray and qRT-PCR analysis. (PDF) [file ppat.1003626.s010.pdf]

Table S2. sX13-regulated genes identified by microarray and qRT-PCR analysis.

Locus[a]	Annotated gene product[b]	4G-motif[c]	Microarray Fold-change (∆*sX13* / wt)[d]		qRT-PCR Fold-change (∆*sX13* / wt)[e]	
			NYG	MMA	NYG	MMA
**Upregulated genes (∆*sX13* / wt)**						
XCV0678	AlgR; two-component system regulatory protein	a,a,a	1.8	–	2.5 ± 0.23	n.t.
XCV0730	Prc; tail-specific protease	–	–	1.6	n.t.	n.t.
XCV0950	conserved hypothetical protein	a,a	1.6	–	n.t.	n.t.
XCV1274	putative secreted protein	a	–	2.7	n.t.	n.t.
**XCV1528**	putative secreted protein	–	–	1.8	n.t.	n.t.
**XCV1626**	peptidyl-prolyl cis-trans isomerase	–	–	1.6	n.t.	n.t.
**XCV1768**	Hfq; host factor-I protein	b	1.6	–	2.4 ± 0.08	1.6 ± 0.31
XCV2041	putative signal transduction protein	a	–	1.9	n.t.	n.t.
XCV2185	conserved hypothetical protein	–	3.7	–	n.t.	n.t.
XCV2186	methyl-accepting chemotaxis protein	a	7.7	–	2.1 ± 0.34	10.2 ± 4.63
XCV2302	conserved hypothetical protein	–	1.5	1.6	n.t.	n.t.
XCV2341	conserved hypothetical protein	–	–	1.5	n.t.	n.t.
XCV2357	conserved hypothetical protein	–	1.7	–	n.t.	n.t.
**XCV2499**	putative membrane protein	a,b	2.0	–	n.t.	n.t.
XCV2565	conserved hypothetical protein	b	–	1.6	n.t.	n.t.
**XCV2608**	type IV secretion system VirJ-like protein	b	1.7	2.5	n.t.	n.t.
XCV2814	PilE; type IV pilus pilin	–	2.8	–	3.3 ± 0.36	n.t.
**XCV2815**	type IV pilus adhesin	b	2.1	–	n.t.	n.t.
XCV2819	type IV pilus assembly protein PilW	a	3.7	4.0	3.4 ± 0.37	5.5 ± 3.0
**XCV2820**	type IV pilus assembly protein PilV	a	2.9	–	n.t.	n.t.
**XCV2821**	type IV pilus assembly protein FimT	a	4.3	7.4	4.2 ± 0.32	3.4 ± 1.27
XCV2917	hypothetical protein	–	2.9	–	n.t.	n.t.
XCV3059	putative secreted protein	b	–	2.1	n.t.	n.t.
XCV3067	PilU; type IV pilus assembly protein ATPase	a	1.8	–	1.7 ± 0.29	n.t.
**XCV3096**	ComEA-related DNA uptake protein	–	–	4.2	n.t.	1.9 ± 0.12
XCV3151	hypothetical protein	b,b	–	1.6	n.t.	n.t.
XCV3230	PilJ; type IV pilus methyl-accepting chemotaxis protein	a	2.2	–	n.t.	n.t.
**XCV3233**	PilG; type IV pilus response regulator	a,b	–	2.0	2.3 ± 0.26	4.1 ± 1.71
**XCV3353**	PilA; type IV pilus assembly protein, major pilin	a	–	2.9	n.t.	n.t.
XCV3371	conserved hypothetical protein	a,b	–	1.8	n.t.	n.t.
**XCV3376**	hypothetical protein	–	2.0	–	n.t.	n.t.
XCV3497	PilQ; type IV pilus assembly protein	a,b	–	1.6	n.t.	n.t.
XCV3498	PilP; type IV pilus assembly protein	–	2.5	–	n.t.	n.t.
XCV3499	PilO; type IV pilus assembly protein	a	2.4	–	n.t.	n.t.
**XCV3500**	PilN; type IV pilus assembly protein	–	2.7	–	2.7 ± 0.16	n.t.
XCV3629	putative amidohydrolase family protein	a	1.9	–	n.t.	n.t.
**XCV3727**	conserved hypothetical protein	a	–	2.3	n.t.	n.t.
**XCV3730**	type IV pilus assembly protein	a,a	2.0	–	n.t.	n.t.
**XCV3927**	putative secreted protein	a	–	1.7	5.6 ± 0.45	8.3 ± 4.54
XCV4099	conserved hypothetical protein	b	–	1.7	n.t.	n.t.
XCV4117	conserved hypothetical protein	b	–	1.5	n.t.	n.t.
**XCV4382**	putative acetyltransferase	–	–	1.5	n.t.	n.t.
**Downregulated genes (∆*sX13* / wt)**						
**XCV0227**	hypothetical protein	–	0.4	–	n.t.	n.t.
XCV0588	putative secreted protein; YceI-like family	–	0.5	–	n.t.	n.t.
XCV1188	conserved hypothetical protein	–	0.5	–	n.t.	n.t.
XCV1315	HrpX; AraC-type transcriptional regulator	–	0.6	–	0.6 ± 0.01	0.7 ± 0.13
XCV1787	predicted ATPase related to phosphate starvation-inducible protein PhoH	–	0.6	–	n.t.	n.t.
XCV1945	methyl-accepting chemotaxis protein	–	0.5	–	n.t.	n.t.
XCV1956	CheA1; chemotaxis protein	–	0.6	–	n.t.	n.t.
XCV1957	CheY; chemotaxis response regulator	–	0.4	–	0.1 ± 0.04	n.t.
XCV1958	putative anti-sigma factor antagonist	–	0.4	–	n.t.	n.t.
XCV2021	FliD; flagellar capping protein	–	0.7	–	n.t.	n.t.
**XCV2022**	FliC; flagellin and related hook-associated proteins	–	0.2	–	0.06 ± 0.03	1.0 ± 0.39
XCV2037	conserved hypothetical protein	–	0.3	–	n.t.	n.t.
XCV2276	hypothetical protein	b	0.6	–	n.t.	n.t.
XCV2282	conserved hypothetical protein	–	0.6	–	n.t.	n.t.
XCV2535	CydA; cytochrome D ubiquinol oxidase, subunit II	a	0.3	–	n.t.	n.t.
XCV2537	putative membrane protein	–	0.1	–	n.t.	n.t.
**XCV3119**	sigma-54 modulation protein	–	0.6	–	n.t.	n.t.
XCV3206	TonB-dependent outer membrane receptor	–	0.4	–	n.t.	n.t.
XCV3572	TonB-dependent outer membrane receptor	a	0.2	–	0.2 ± 0.04	0.9 ± 0.24
**XCV4119**	putative secreted protein	–	0.2	–	n.t.	n.t.
**XCVd0093**	putative secreted protein	–	0.5	–	n.t.	n.t.

Locus[a]	Annotated gene product[b]	4G-motif[c]	Microarray Fold-change (Δ*sX13* / wt)[d]		qRT-PCR Fold-change (Δ*sX13* / wt)[e]	
			NYG	MMA	NYG	MMA
**Additional genes tested by qRT-PCR**						
XCV0173	putative secreted protein	a,b,b,b	–	–	1.9 ± 0.19	0.8 ± 0.26
**XCV0612**	ATPase of the AAA+ class	a	–	–	1.0 ± 0.06	0.8 ± 0.26
XCV1533	AsnB2; asparagine synthase	b	–	–	1.0 ± 0.04	1.0 ± 0.17
XCV3232	PilH; type IV pilus response regulator	a	–	–	2.2 ± 0.07	1.9 ± 0.67
XCV3573	putative transcriptional regulator, AraC family	a	–	–	0.2 ± 0.11	n.t.
XCV0324	type III effector protein XopS	–	–	–	0.6 ± 0.05	n.t.

[a], bold letters indicate genes with known TSS [1]. [b], refers to Thieme *et al.* (2005) [2]. [c], presence of a 4G-motif within the 5'-UTR or 100 bp upstream of translation start codon if TSS is unknown (a) and within 100 bp downstream of the start codon (b) (see Figure S4). [d], genes not detected as expressed are marked with –. [e], values represent mean fold-change and standard deviation (see Figure 4); n.t. - not tested.

References

1. Schmidtke C, Findeiss S, Sharma CM, Kuhfuss J, Hoffmann S, et al. (2012) Genome-wide transcriptome analysis of the plant pathogen *Xanthomonas* identifies sRNAs with putative virulence functions. Nucleic Acids Res 40: 2020-2031.
2. Thieme F, Koebnik R, Bekel T, Berger C, Boch J, et al. (2005) Insights into genome plasticity and pathogenicity of the plant pathogenic bacterium *Xanthomonas campestris* pv. vesicatoria revealed by the complete genome sequence. J Bacteriol 187: 7254-7266.
